# Reduced Neutrophil Extracellular Trap (NET) Formation During Systemic Inflammation in Mice With Menkes Disease and Wilson Disease: Copper Requirement for NET Release

**DOI:** 10.3389/fimmu.2019.03021

**Published:** 2020-01-15

**Authors:** Iwona Cichon, Weronika Ortmann, Aleksandra Bednarz, Malgorzata Lenartowicz, Elzbieta Kolaczkowska

**Affiliations:** ^1^Department of Experimental Hematology, Institute of Zoology and Biomedical Research, Jagiellonian University, Kraków, Poland; ^2^Department of Genetics and Evolutionism, Institute of Zoology and Biomedical Research, Jagiellonian University, Kraków, Poland

**Keywords:** endotoxemia, sepsis, neutrophils, neutrophil extracellular traps, trace elements, copper, Menkes disease, Wilson disease

## Abstract

Neutrophil extracellular traps (NETs) contribute to pathological disorders, and their release was directly linked to numerous diseases. With intravital microscopy (IVM), we showed previously that NETs also contribute to the pathology of systemic inflammation and are strongly deposited in liver sinusoids. Over a decade since NET discovery, still not much is known about the metabolic or microenvironmental aspects of their formation. Copper is a vital trace element essential for many biological processes, albeit its excess is potentially cytotoxic; thus, copper levels are tightly controlled by factors such as copper transporting ATPases, ATP7A, and ATP7B. By employing IVM, we studied the impact of copper on NET formation during endotoxemia in liver vasculature on two mice models of copper excess or deficiency, Wilson (ATP7B mutants) and Menkes (ATP7A mutants) diseases, respectively. Here, we show that respective ATP7 mutations lead to diminished NET release during systemic inflammation despite unaltered intrinsic capacity of neutrophils to cast NETs as tested *ex vivo*. In Menkes disease mice, the *in vivo* effect is mostly due to diminished neutrophil infiltration of the liver as unmutated mice with a subchronic copper deficiency release even more NETs than their controls during endotoxemia, whereas in Wilson disease mice, excess copper directly diminishes the capacity to release NETs, and this was further confirmed by *ex vivo* studies on isolated neutrophils co-cultured with exogenous copper and a copper-chelating agent. Taken together, the study extends our understanding on how microenvironmental factors affect NET release by showing that copper is not a prerequisite for NET release but its excess affects the trap casting by neutrophils.

## Introduction

The capacity to cast neutrophil extracellular traps (NETs) by neutrophils was described 15 years ago, yet not all aspects or mechanisms of their release were revealed thus far (1). NETs are still being studied mostly using isolated neutrophils, but it seems critical to follow their release *in vivo* where NET formation might be affected by additional factors such as the presence of other cell populations (specific to the organ as well as other leukocytes) and microenvironment conditions such as local plasma content. In particular, the immunometabolic requirements for NET release are still ambiguous, including requirement for some macro- and micronutrients. Thus, far, only zinc dependency of NETs was reported (2), whereas impact of iron was inconclusive as various chelating agents were either stimulating or inhibiting NET formation (3, 4). NETs are structures released by highly activated neutrophils whose backbone consists of DNA decorated with nuclear histones and granular proteins such as antimicrobials (e.g., defensins) and proteases (e.g., neutrophil elastase, NE), but also cytoplasmic and cytoskeletal proteins (1, 5). While originally NETs were believed to be beneficial for the host defense via their capacity to capture and immobilize pathogens (1, 6), it was subsequently recognized that they can also cause bystander damage or contribute to disease outbreak or relapses (7–9). This “Dr. Jekyll and Mr. Hyde phenotype” can be attributed to early and late stages of inflammation, respectively. One of conditions in which release of NETs goes from beneficial [early blood bacterial trapping (10)] to detrimental [delayed organ damage (7)] is sepsis and endotoxemia (6). As systemic inflammation is associated with high mortality, independently of the causative pathogen or just the presence of lipopolysaccharide (LPS)/endotoxin (11), this calls for new therapeutic strategies but first requires gaining detailed knowledge on the mechanisms of NET release. Recent studies have shown liver dysfunction as an early event in systemic inflammation (12), and intravital microscopy revealed that during sepsis or endotoxemia, NETs are released robustly into liver sinusoids of mice (6, 7, 10). Beyond systemic inflammation, there are multiple conditions in which liver is strongly affected, and some of them are connected with excess/deficiency of microelements (13–16).

Metal ions play an essential role in many biological processes acting as chemical catalyzers, structure stabilizers, gene regulators, and signaling molecules (17). This also includes immune processes as metal ions are essential for both leukocytes and pathogens (18) and transition metals/trace elements important in immunity include Fe, Zn, Mn, and Cu (18, 19). Interestingly, when PMA (a phorbol ester) was used to induce NETs, neutrophils contained void vacuoles with visibly reduced concentrations of Ca, Fe, Zn, and Cu but NETs themselves did not contain Cu although they did Ca, Fe, P, and S (20).

At the cellular level, copper serves as a cofactor in multiple proteins due to its redox ability (19, 21, 22). Its uptake (both dietary and peripheral distribution) occurs via membrane copper transporters, including CTR1 (23). Copper is important for the immune system function as macrophage and lymphocyte activities are diminished when it is deficient (24). As to neutrophils, their maturation is altered in copper-deficient individuals. In humans, neutropenia accompanying copper deficiency results from arrest of maturation of neutrophils (promyelocytes/metamyelocytes ratio is altered) (25) but most probably also from their impaired release from the bone marrow and decreased life span of circulating ones (26). Furthermore, anti-neutrophil antibodies were detected in the serum of copper-deficient patients, suggesting a possible reason for (partial) depletion of neutrophils (27). On the other hand, the excess of copper is highly toxic because this element is a potent inducer of reactive free radicals that can cause oxidative damage to proteins, lipids, and nucleic acids (21). Therefore, numerous mechanisms were developed to control copper levels, and they include P-type ATPases (ATP7A and ATP7B), which are involved in copper efflux and intracellular sequestration (21, 28). Mutations in *Atp7a* gene result in depletion of copper in the liver due to impaired intestinal copper intake while *Atp7b* mutations lead to systemic accumulation of copper due to impaired excretion by the liver (14, 16, 28–30). Therefore, we undertook the current study to verify the impact of copper excess and deficiency on NET release into liver sinusoids during systemic inflammatory reaction. The facts that directly prompted us to the investigation were the following: (i) neutrophil status is affected by the lack of copper (26), (ii) copper is released during NET formation (20), (iii) excess of copper affects the liver (31), (iv) NETs are strongly deposited in the liver during systemic inflammation (7, 10), and (v) deficiency of other trace elements was shown to affect NET release (2, 3). Since we aimed to primarily study NETs *in situ* in the liver vasculature, where they are formed during systemic inflammation, we used two mouse models of human genetic disorders in which copper homeostasis is disrupted, Menkes disease and Wilson disease. In addition, liver is one of the organs that is mostly affected by genetic copper excess (Wilson disease) (14) or marginal copper deficiency (leading to non-alcoholic fatty liver disease, NAFLD) (32).

Wilson disease patients present with progressive liver damage as well as neurological disorders and psychiatric symptoms (31). In toxic milk mice (tx-J) that serve as the disease model, the gene encoding ATP7B is affected. This protein serves as a copper-transporting molecule that, at high intracellular copper concentrations, effluxes its excess. Thus, if ATP7B is malfunctioning, this process is impaired as well as subsequent copper excretion into the bile, which results in copper accumulation in the liver, brain, and, to a lower extent, in other tissues (29, 30). By 6 months of age, copper concentration can be over 50-fold more than that of a normal adult animal (33).

On the other hand, mutations in the human *ATP7A* gene lead to a neurological disorder called Menkes disease, which usually results in mortality at 3–5 years of age in its classical form (16). The mouse ATP7A protein is encoded by the X-linked *Atp7a* gene, and among its several reported mutations in mice, one (Atp7a^mo-*ms*^) results in the *mottled* phenotype resembling Menkes disease and affects mosaic mutant males (15, 34). In these mice, similarly to patients with Menkes disease, copper accumulates in the small intestine (trapped in enterocytes) and kidneys, while liver, brain, and heart display its deficiency (34). Additionally, as in the classical form of Menkes disease, the mosaic mutation of *Atp7a* leads to early death on about day 16. The phenotype can be partially reversed by regular s.c. copper (II) chloride injections since the second day of life till day 45 (34, 35). Mice still display symptoms of Menkes disease, and their copper levels are decreased.

With the application of intravital microscopy, herein we reveal that NET formation in the vasculature of endotoxemic liver is impaired in mice with genetic mutations leading to either deficiency or excess of copper. The two mutations affect *Atp7a* and *Atp7b*, respectively, and we demonstrate that the products of both genes are expressed by some neutrophils. Polymorphonuclear leukocytes of animals with Wilson disease (Atp7btx-J/J) casted less NETs despite more profound infiltration of the liver. *Ex vivo* studies confirmed that copper concentration beyond its serum levels inhibits NET formation and in higher concentration kills the cells. On the other hand, in Menkes disease mice (Atp7a^mo-*ms*^), weaker NET formation correlated with lower neutrophil numbers. However, in mice with subchronic copper deficiency (inbred mice treated for 8 days with a copper chelator), despite decreased neutrophil counts, NET formation was even increased. This indicates that copper might not be required for NET casting or even interfere with it whereas in *Atp7a* mutants, additional traits are present and we discuss them. Taken together, we demonstrate that while copper does not seem to be a prerequisite for NET formation, its high levels affect this process and directly kill neutrophils.

## Materials and Methods

### Mice

All animals were maintained in the Animal Unit of the Institute of Zoology and Biomedical Research, Jagiellonian University. Mice were housed under standardized conditions of temperature (21–22°C) and illumination (12 h light/12 h darkness) with free access to tap water and pelleted food (Labofeed H diet, Kcynia, Poland). As a reference inbred strain, age-matched C57Bl/6J male mice were used. They were purchased from Charles River (Germany) via AnimaLab (Poland). All experimental animal protocols were approved by the Local Ethical Committee No. II in Kraków (293/2017 and 293A/2018) and were in compliance with the EU Animal Care Guidelines. Main murine strains used in experiments were *Atp7a* mosaic mutant mice and *Atp7b* toxic milk mice and their respective littermates.

### *Atp7a* Mosaic Mutant Mice (The Menkes Disease Model)

Mice were bred in the Department of Genetics and Evolutionism, Jagiellonian University, and derived from a closed outbred colony. In the present study we used mosaic mutant males, which exhibit lethal phenotype about day 16 after birth and age-matched wild type males. Mosaic missense mutation (^*ms*^/^−^) consists of G to C nucleotide change in the exon 15 of *Atp7a* gene resulting in an arginine to proline substitution in the 6th highly conserved transmembrane domain of ATP7A protein (15). Mosaic mutant males (^*ms*^/^−^) were obtained by mating heterozygous (^*ms*^/^+^) females with normal (^+^/^−^) males. To rescue the otherwise lethal phenotype, mice were receiving subcutaneous injections of 50 μl of 0.01% CuCl_2_ solution (5 μg Cu/g body weight) every 2 days from day 2 to day 45 after birth. Very few pups survive. In the present study, we used 3-month-old males that could still survive without further copper supplementation and reach maturity (36).

### *Atp7b* Toxic Milk Mice (The Wilson Disease Model)

The mutant C3HeB/FeJ Atp7btx-J/J and control C3HeB/FeJ mice were purchased from The Jackson Laboratory (Bar Harbor, ME, USA) and bred in the Department of Genetics and Evolutionism, Jagiellonian University. The tx-J mutants have a genetic defect that originated due to a G to A base substitution at the position 2,135 in the exon 8 of the *Atp7b* gene; this caused a Gly712Asp missense mutation in the second putative membrane-spanning domain of the ATP7B protein (37). Homozygous mutants (tx-J/tx-J) were obtained by mating heterozygous (tx-J/+) females with heterozygous (tx-J/+) males, and tail biopsy was obtained from each mouse to check for *Atp7b* mutation. Experiments were performed on 7/8-month-old male mice. The age was adjusted in such a way that a 6-month point of very high copper concentration was reached (33).

### Antibodies and Dyes for IVM

For intravital microscopy, the following antibodies were used, Alexa Fluor 647 anti-neutrophil elastase (clone G-2, Santa Cruz Biotechnology), Brilliant Violet 421 anti-Ly6G (1A8, BioLegend), PE anti-F4/80 (clone BM8, eBioscience), or Alexa Fluor 488 anti-F4/80 (clone BM8, Invitrogen), and PE anti-CD49b (clone HMα2, BioLegend). Sytox green (the DNA dye) was purchased from Invitrogen. All antibodies were injected i.v. via the jugular vein ~20 min prior to intravital imaging. Sytox green was administrated during imaging as it stains DNA instantly.

### Induction of Systemic Inflammation/Endotoxemia

Mice (C3HeB/FeJ Atp7btx-J/J, C3HeB/FeJ, Atp7^*amo-ms*^, outbreds, C57Bl/6J) were i.p. injected with 1 mg/kg b.w. LPS (*Escherichia coli* serotype 0111:B4; Sigma-Aldrich), and they were subjected to intravital imaging 24 h post LPS injection. Some animals were left untreated. In some experiments, prior to endotoxemia induction, C57Bl/6J were injected i.p. with 5 mg/kg b.w of copper chelator tetrathiomolybdate (TTM, Sigma-Aldrich) every 24 h for 8 days.

### Preparation of the Mouse Liver for Intravital Microscopy

Mice were anesthetized with a mixture of ketamine hydrochloride (200 mg/kg b.w.; Biowet Pulawy) and xylazine hydrochloride (10 mg/kg b.w.; aniMedica). After anesthesia, cannulation of the right jugular vein was performed for the supply of the anesthetics and for injection of antibodies or other reagents. Preparation of the liver for intravital imaging was performed as previously described by Kolaczkowska et al. (7). Briefly, a midline incision followed by a lateral incision along the costal margin to the midaxillary line was performed to expose the liver. The mouse was placed in a right lateral position, and ligaments attaching the liver to the diaphragm and the stomach were cut, thus allowing the liver to be externalized onto an imaging board covered with a saline-soaked kimwipe tissue. Then, a coverglass was placed on the left liver lobe and the space underneath the coverglass was filled with saline (it was constantly refilled to keep the tissue moist). The animal positioned on the imaging board was subsequently placed under the upright microscope.

### Spinning Disk Confocal Intravital Microscopy

The mouse liver was visualized with a ZEISS Axio Examiner.Z1 upright microscope equipped with a metal halide light source (AMH-200-F6S; Andor, Oxford Instruments) with motorized 6 position excitation filter wheel and laser-free confocal spinning disk device (DSD2; Andor, Oxford Instruments) with ZEISS EC Plan-NEOFLUAR 10×/0.3 and/or ZEISS EC Plan-NEOFLUAR 20×/0.5 air objective. Four excitation filters were used (DAPI: 390/40 nm; GFP: 482/18 nm; RFP: 561/14 nm; Cy5: 640/14 nm) and visualized with the appropriate emission filters (DAPI: 452/45 nm, exposure time 500 ms; GFP: 525/45 nm, exposure time 500 ms; RFP: 609/54 nm, exposure time 500 ms; Cy5: 676/29 nm, exposure time 250 ms). The 5.5 megapixel sCMOS camera (Zyla 5.5; Andor, Oxford Instruments) was used for fluorescence detection. An iQ 3.6.1 acquisition software (Andor, Oxford Instruments) was used to drive the microscope.

### NET Formation Analyses

Fluorescence imaging of NET components was performed with intravital immunofluorescence analysis. NETs were visualized by co-staining of neutrophil elastase (anti-NE antibodies; 1.6 μg) and extracellular DNA (Sytox green; 0,1 mM in saline). NETs were quantified with SD-IVM using previously published methodology (7, 38). In brief, images were acquired as *z* stacks of *xy* planes (1 mm intervals) from the bottom to the top of sinusoids in each field of view using a 20× objective lens, and saved as extended focus images in tiff format. Images from individual color channels were exported and analyzed in ImageJ software (NIH). The intensity of elastase staining was analyzed so that differences in background fluorescence between experiments and antibody lots could be accounted for and background autofluorescence could be eliminated, contrast was adjusted to minimize autofluorescent background staining, and a minimum brightness threshold was set to yield only positive staining. The same contrast and threshold values were applied to all images from all mice strains and groups within the experiment. Thresholded images were converted to binary (black and white), and the area per field of view (FOV) covered by positive fluorescence staining (black) was calculated with ImageJ software. Data are expressed as the percentage of area in each FOV covered by positive fluorescence staining. In *ex vivo* studies on isolated neutrophils, the cells were incubated on coverglasses, and then subsequently immunocytostained for the presence of NETs (as described below). NETs stained on coverglasses were visualized using the same microscope. Briefly, in ImageJ, images were converted to a grayscale (8-bit type) and thresholded to black and white to estimate the positive signal of extDNA/NET (black).

### Neutrophil, Kupffer Cell, and Platelet Counts

Neutrophils were visualized with anti-mouse Ly6G antibodies (0.4 μg/mouse), Kupffer cells (KCs) were stained with anti-F4/80 antibodies (0.4 μg for PE or 3 μg for Alexa Fluor 488/mouse), and platelets were stained with anti-CD49b antibodies (0.6 μg/mouse). Neutrophils and KCs were counted per 20× FOV, minimum 4 FOV from each mouse. The area covered by platelets was estimated as described previously (7, 38) and above for NETs, on images acquired as *z* stacks of *xy* planes (1 mm intervals) from the bottom to the top of sinusoids and is expressed as the percentage of area in each FOV covered by positive fluorescence staining.

### 3D Reconstruction of Liver Cross-Sections

Overview of mouse livers was established on a side view of a 3D reconstruction (IMARIS software, Bitplane, Oxford Instruments) of a series of optical cross-sections (*z* stacks). In brief, a series of *z* stacks through the liver was performed with a z-step of 1 μm approximately through 50 *z* planes. Then, liver structure was reconstructed with IMARIS software with DAPI, RFP, and Cy5 channels, for neutrophils, KCs, and neutrophil elastase, respectively. Additionally, on some acquired *z* stacks, red signal (KC staining) was made semi-transparent to expose the blue signal (neutrophil staining) present inside.

### Isolation of Mouse Neutrophils From the Bone Marrow

Bone marrow neutrophils were isolated as described previously (39). Briefly, mice were euthanized and the femurs and tibias were removed. The ends of the bones were resected and the bone marrow was removed by flushing with 10 ml of ice-cold HBSS(–) (w/o Ca^2+^ and Mg^2+^; Lonza Bioscience). The bone marrow was then suspended by drawing it through a 20-gauge needle. Marrow cells were then pelleted in a centrifuge (1,300 rpm, 4°C, 6 min) and after that resuspended in 5 ml of 0.2% NaCl for about 30 s for hypotonic lysis of the red blood cells followed by the addition of 5 ml of 1.6% NaCl. After immediate centrifugation (1,400 rpm, 4°C, 7 min), the cell suspension was placed over a discontinuous Percoll gradient (GE Healthcare) consisting of 78, 69, and 52% layers diluted in HBSS(–). The cell suspension was spun at 2,600 rpm, 4°C, for 30 min. Neutrophils localized to a band between the 78 and 69% layers were collected with a transfer pipette and washed in HBSS(–) (1,500 rpm, 4°C, 6 min), and the cell pellet was suspended in HBSS(+) (HBSS with Ca^2+^ and Mg^2+^) (Lonza Bioscience).

### *Ex vivo* Neutrophil Viability, Counts, and Adhesion

The purity of isolated neutrophils was over 99%, and neutrophil viability was over 98% in each experiment. To estimate viability, cells were stained with Trypan blue dye (Sigma-Aldrich) and counted in the Bürker hemocytometer. The numbers and purity were assessed by Türk solution (0.01% crystal violet in 3% acetic acid) staining. The viability was double-checked with PrestoBlue® (Invitrogen) assay, which yielded the same results. The ability of the cells to adhere to cell culture plate after LPS (75 μg/ml) stimulation was tested using the crystal violet test (CV). After incubation, supernatants with non-adherent cells were removed, whereas adhering cells were fixed with absolute methanol (RT, 10 min). After fixation, methanol was removed and crystal violet solution (25 mg in 5 ml 20% methanol; Avantor) was added to each well (RT, 5 min). Following incubation, the plate was gently washed in running tap water and air-dried. To recover crystal violet, 100 μl of 100% methanol was added to each well and the plate was agitated for 10 min. The optical density (O.D.) was measured in a spectrofluorometer (Infinite 200 Pro, Tecan, Austria, Gmbh) at 570 nm.

### *Ex vivo* Evaluation of Reactive Oxygen Species/Respiratory Burst

Content of reactive oxygen species (ROS) in LPS-stimulated neutrophils was measured by two methods, NBT test, and fluorescent DCF-DA assay. In the latter test, the cell-permeable fluorogenic probe 2′,7′-Dichlorofluorescin Diacetate (DCF-DA; Sigma-Aldrich) was added to each well at a final concentration of 25 μM, 30 min after neutrophil stimulation with LPS (75 μg/ml). For the positive control, 1 mM H_2_O_2_ was used. After 30 min of incubation (37°C, 5% CO_2_) the plate was centrifuged (3,000 rpm, 5 min, RT) and cell pellets were rinsed in 100 μl of PBS. Then, the fluorescence intensity was measured in a spectrofluorometer (Infinite 200 Pro, Tecan, Austria, Gmbh) at 485(20)/535(25) nm. The respiratory burst was measured with nitroblue tetrazolium assay (NBT). Briefly, neutrophils were stimulated with LPS (75 μg/ml) and 1 h before the end of the incubation period, NBT solution (10 mg/ml in dH_2_O; Sigma Aldrich, St. Louis, MO) was added to each well and incubated for 1 h (37°C, 5% CO_2_). Following the incubation, supernatants were removed and the cells were fixed with absolute methanol for 15 min. They were subsequently washed twice with 70% methanol, air-dried, and solubilized (in 120 μl of 2 M potassium hydroxide and 140 μl of dimethyl sulfoxide) to release formazan deposits. The O.D. was measured in a spectrofluorometer (Infinite 200 Pro, Tecan, Austria, Gmbh) at 595 nm.

### *Ex vivo* Studies on NET Release by Neutrophils

Neutrophils suspended in HBSS(+) were seeded in wells of either 96- or 24-well plates (Nest; Thermo Scientific, respectively). They were let to adhere for 30 min and then were stimulated for 6 h with LPS (75 μg/ml), various concentrations of copper (II) chloride (Cu 0.25, 0.5, 1, 2, 3, 130, and 200 μg per ml; Sigma-Aldrich), copper chelator tetrathiomolybdate (TTM 10 μM), or a mixture of Cu and TTM. In some experiments, phorbol 12-myristate 13-acetate (PMA, 50 nM/3 h; Sigma-Aldrich) was used as a positive control for NET formation. After incubation, cell viability was verified with PrestoBlue®. Alternatively, immediately after incubation, the cells were carefully fixed in sequence of 1, 2, and 3% paraformaldehyde in PBS for 2, 10, and 20 min, respectively, to not disrupt formed NETs and washed in PBS. Neutrophils seeded in 24-well plates were used for immunocytochemistry.

### Immunocytochemical Staining of NETs, CTR1, and ATP7A/B in Neutrophils

After incubation with stimulants, neutrophils were immediately fixed in paraformaldehyde as described above. Extracellular staining [for citrullinated histones H3 (citH3) and extracellular DNA (extDNA)]: prior to staining, coverglasses were washed two times for 5 min in PBS, and then incubated in blocking solution (3% BSA in PBS) (BSA, bovine serum albumin, Sigma-Aldrich) for 45 min at RT. Subsequently, coverglasses were soaked with rabbit polyclonal anti-histone H3 (citrulline R2 + R8 + R17) antibodies diluted 1:200 in 1% BSA/PBS (Abcam) and incubated overnight at 4°C in a humid chamber. The slides were then washed in PBS and incubated with Cy3-conjugated goat anti-rabbit IgG (H+L) antibody (diluted 1:300 in PBS/1% BSA, Jackson Immunoresearch) for 1 h at RT. At the end of the procedure, Sytox green was added to stain for extDNA (5 μM). After washing in PBS the coverglasses were mounted with VECTASHIELD Mounting Medium (Vector Laboratories). Intracellular staining (CTR1, ATP7A, ATP7B): prior to staining, cells seeded on coverglasses were permeabilized by bathing in TBS (Triton X-100, Na_2_HPO_4_ × 12H_2_O, Na_2_HPO_4_ × 1H_2_O, BSA, NaCl, dH_2_O) for 5 min. Non-specific antibody binding was blocked by incubation with 3% BSA/PBS for 45 min. Subsequently, the cells were labeled with rabbit polyclonal antibodies anti-ATP7A (diluted 1:100; Abcam) or anti-ATP7B (diluted 1:100; GeneWay) or rabbit polyclonal anti-SLC31A/CTR1 (diluted 1:250; Novus Biologicals) and incubated overnight at 4°C in a humid chamber. The slides were then washed in PBS and incubated with the secondary antibody goat anti-rabbit IgG–H&L (Cy3) (diluted 1:100) for 1 h at RT. After the coverglasses were washed in PBS, they were mounted with VECTASHIELD Mounting Medium. Fluorescent signal was detected with a ZEISS Axio Examiner.Z1 upright microscope equipped with confocal spinning disk device DSD2.

### Measurement of Copper Content in Livers and Plasma

The level of copper in livers obtained from mutants and the wild-genotype control male mice was measured by atomic absorption spectrophotometry (AAS). The liver samples were weighed and digested in 2 ml of boiling Suprapur-grade nitric acid (Sigma-Aldrich). After being cooled down to RT, each sample was suspended in 10 ml of deionized water. Reference material samples were prepared in a similar manner. The copper concentration was measured using the graphite furnace AAS technique (AAnalyst 800, Perkin-Elmer). Moreover, three samples of nitric acid were used as blanks. In addition, three samples of a standard reference material, Cu = 189 ± 4 mg/kg, were analyzed for normalization of the obtained data.

### Biochemical Assessment of Liver Injury

Blood was collected by cardiac puncture into a heparinized syringe. Samples were centrifuged at 1,200 *g* for 10 min for the retrieval of plasma. Subsequently, mice were euthanized and their livers were collected and homogenized. Samples were analyzed for ALT activity as per the manufacturer's protocol (Sigma-Aldrich).

### Statistics

All data are presented as mean values ± SD. Data were compared either by unpaired two-tailed Student's *t* test or one-way analysis of variance with Bonferroni multiple comparisons *post hoc* test. Statistical significance was set at *P* < 0.05.

## Results

### NET Release by Neutrophils in Septic Mice With Menkes and Wilson Diseases

**Figure 1:**
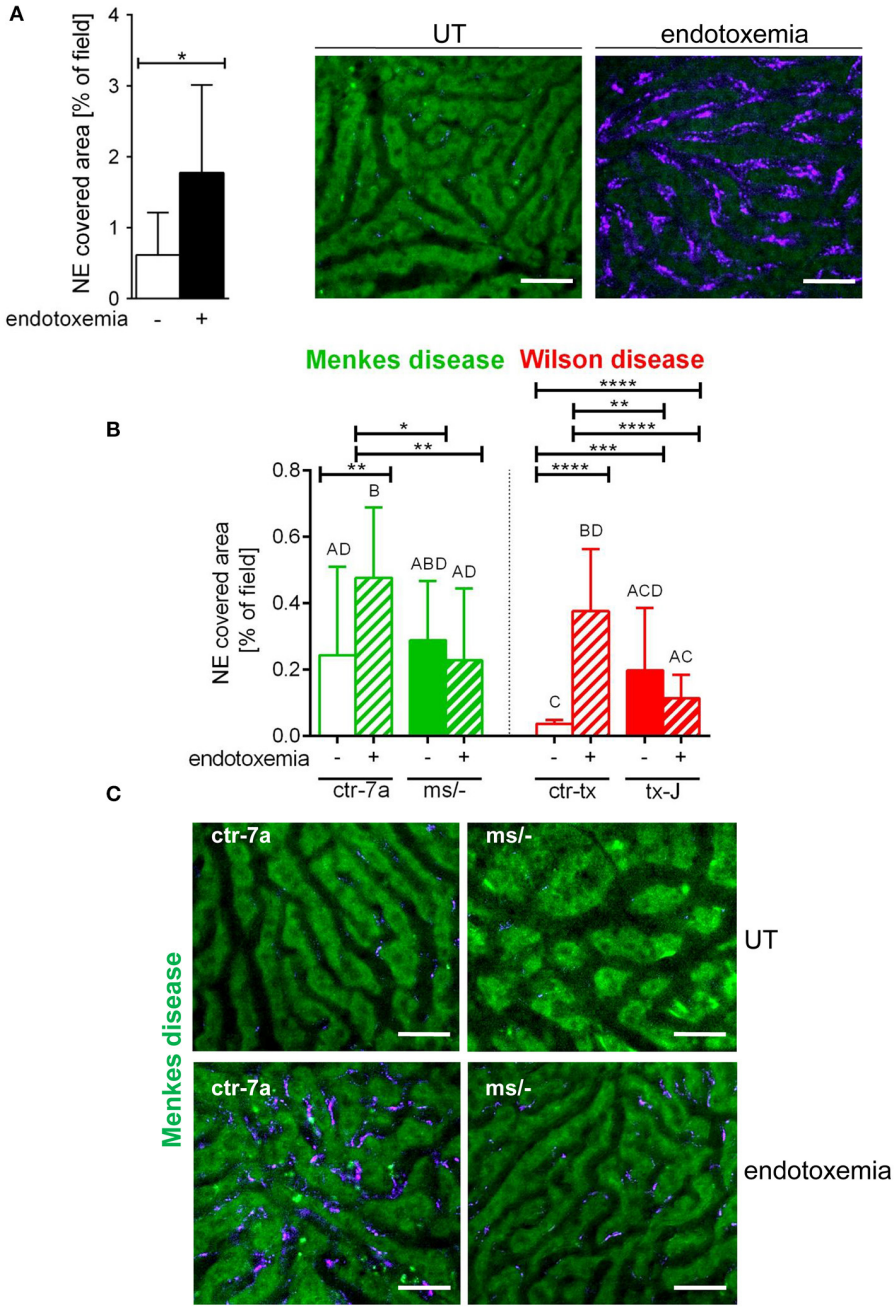
Deposition of neutrophil extracellular traps (NETs) in liver sinusoids during endotoxemia in C57Bl/6J mice **(A)** and in animals with Wilson (tx-J) and Menkes (^ms/−^) diseases and their respective controls — ctr-tx and ctr-7a **(B,C)**. NET formation was quantified as the percentage of area covered by neutrophil elastase (NE) in each analyzed field of view (FOV) (mice *n* = 3/group). Asterisks indicate significant differences using unpaired two-tailed Student's *t* test (*P ≤ 0.05, **P ≤ 0.01, ***P ≤ 0.001, ****P ≤ 0.0001) in **(A,B)** or one-way ANOVA (*post hoc* Bonferroni) test in **(B)**; different letters indicate statistically significant differences between groups. Representative pictures of NE deposition in livers of C57Bl/6J mice are presented in **(A)** and of Menkes disease mice and their littermates in **(C)** (images of Wilson disease mice and their controls are presented in Supplementary Figure 1). On images, autofluorescent hepatocytes (green) can be observed, and between them, sinusoids are seen as black areas. In the latter structures, NE signal is deposited along endothelium (violet). UT, untreated. The scale bar indicates 50 μm.

**Figure 2:**
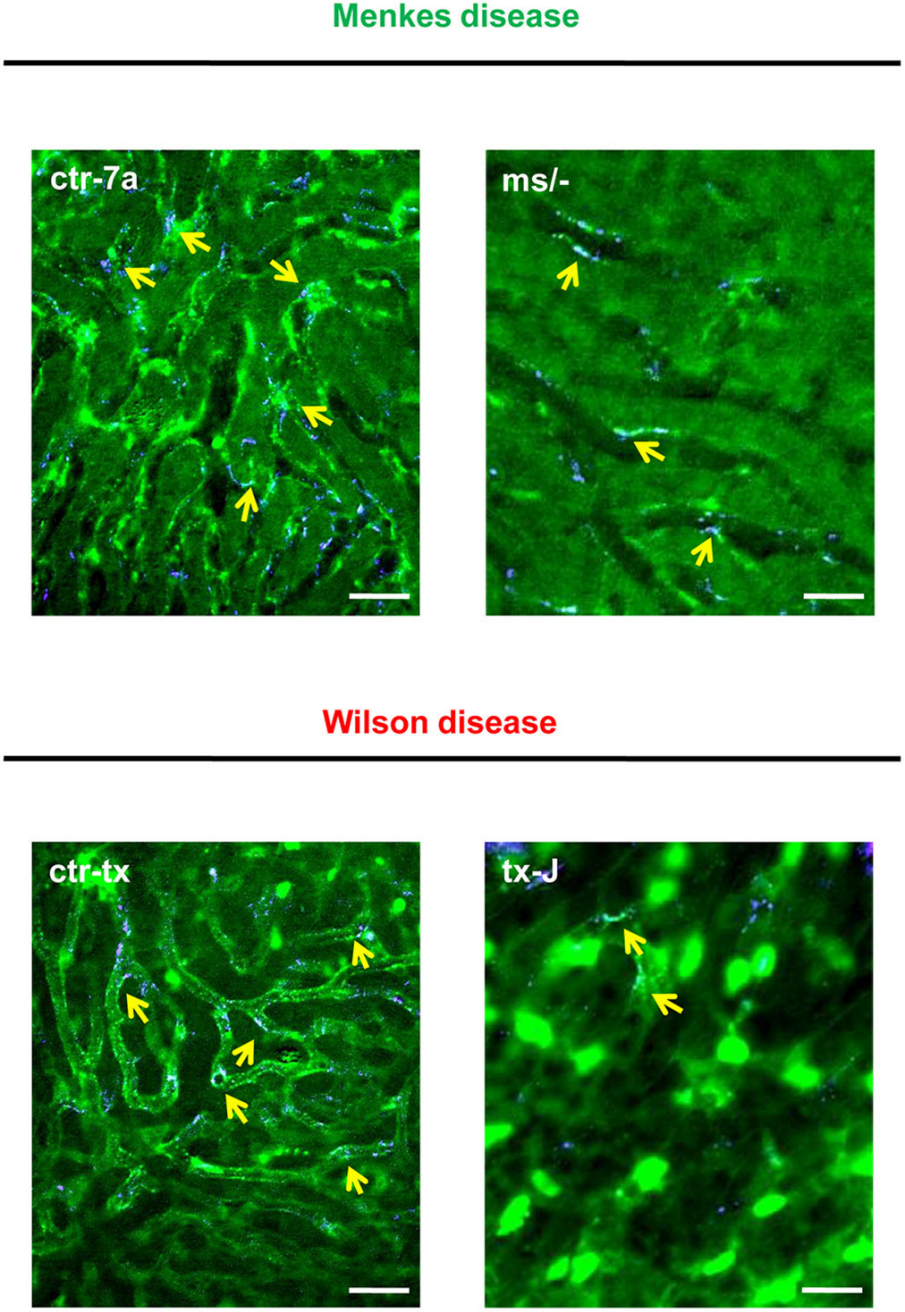
Detection of NET components in the liver vasculature of endotoxemic Wilson (tx-J) and Menkes (^ms/−^) disease mice and their respective controls (ctr-tx and ctr-7a). On images, autofluorescent hepatocytes (dim green) as well as Sytox green^+^ extracellular DNA (bright green) and NE signal (violet) are seen. Overlay of extDNA and NE is indicative of NETs (yellow arrows). The scale bar indicates 50 μm.

NETs are hardly present in the vasculature of untreated mice (6, 7, 10), and it was also the case in this study (Figure 1 and Supplementary Figure 1). During systemic inflammation, all parameters were studied 24 h post LPS administration. *In vivo* NET release was thus far studied mainly in C57Bl/6J mice (7, 10, 40), and for this reason, we compared the release of the traps by *Atp7a/b* mutants and their unmutated counterparts to the above inbred strain (Figures 1A–C). Littermates of the mutant mice — outbred (ctr-7a) for ^ms/−^ animals with Menkes disease and C3HeB/FeJ (ctr-tx) for tx-J mice with Wilson disease — did produce NETs upon endotoxemia, but, significantly <C57Bl/6J mice (Figure 1A vs. Figure 1B). To detect NETs in the liver, mice were injected with fluorescent Sytox green to stain extDNA and Alexa Fluor 647 labeled antibodies directed against neutrophil elastase. Overlaying signals of the nuclear and granular components of neutrophils laying along sinusoid walls were considered indicative of NETs as previously published (6, 7, 10). Accordingly, Figure 2 presents exemplary images of NETs formed in liver sinusoids of ^*ms*^/^−^ and tx-J mice and their counterpart controls. To quantify NET release, the area covered by neutrophil elastase was estimated with ImageJ as described previously (6, 7, 10). Importantly, in both Menkes and Wilson disease endotoxemic mice, NET formation was significantly lower in comparison to their respective unmutated controls with provoked inflammation (Figure 1C, Supplementary Figure 1, and Supplementary Movies 1, 2). In fact, upon LPS stimulation, no increase in NET release was observed in either of the mutants when compared to their healthy counterparts.

### Neutrophil Influx Into Livers of Septic Mice With Menkes and Wilson Diseases

**Figure 3:**
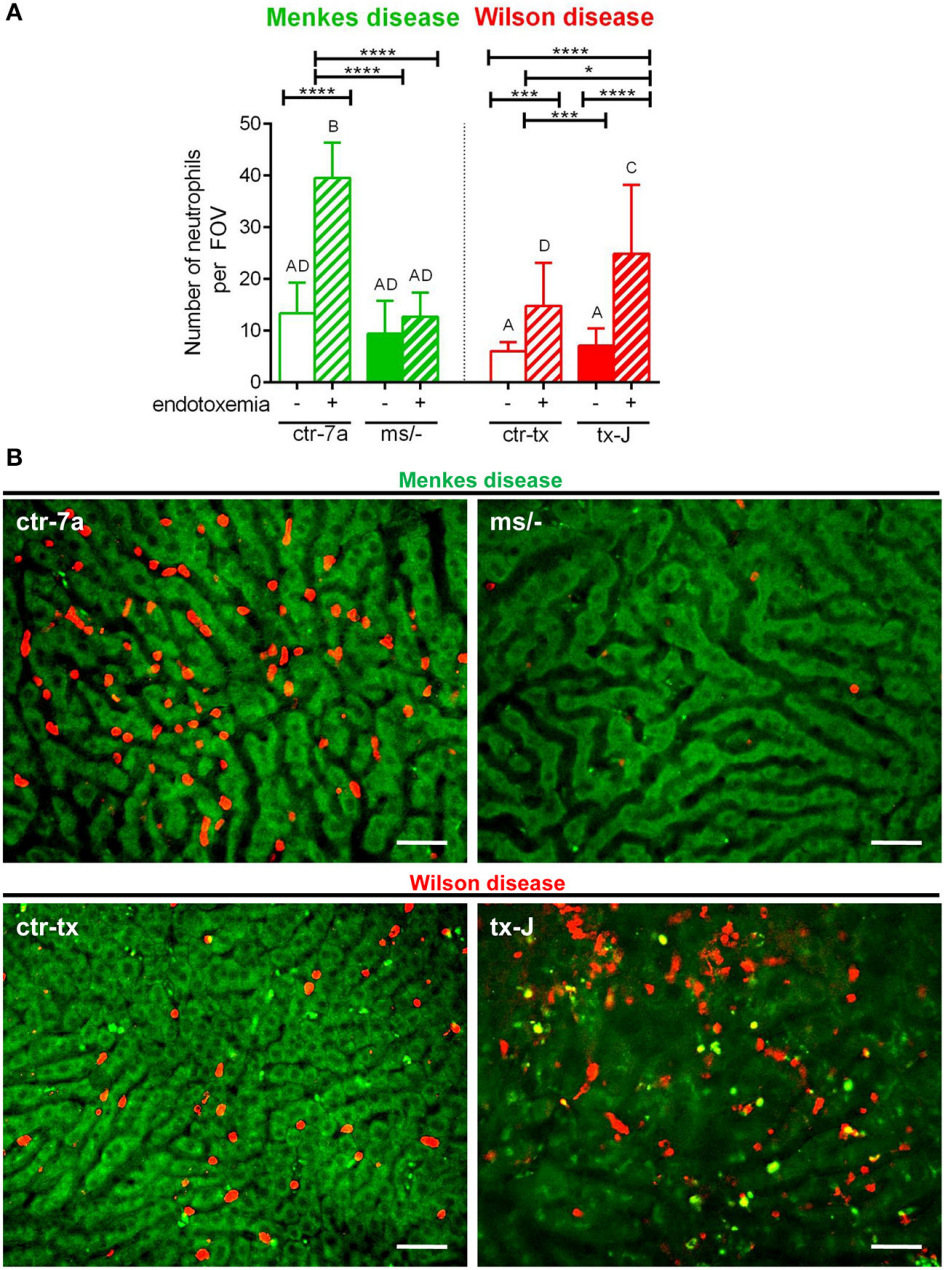
The presence of neutrophils in livers of Wilson (tx-J) and Menkes (^ms/−^) disease mice and their respective controls (ctr-tx and ctr-7a) with systemic inflammation. Neutrophils were counted per field of view (FOV; *n* = 3/group) **(A)**. Asterisks indicate significant differences using unpaired two-tailed Student's *t* test (*P ≤ 0.05, ***P ≤ 0.001, ****P ≤ 0.0001) and one-way ANOVA (*post hoc* Bonferroni) test (different letters indicate statistically significant differences between groups). Representative images revealing neutrophil deposition in liver sinusoids are presented in **(B)**. On images, autofluorescent hepatocytes (green) can be observed, and between them, sinusoids are seen as black areas. In the latter structures, neutrophils are deposited (false red color; Brilliant Violet 421 anti-Ly6G antibody). The scale bar indicates 50 μm.

**Figure 4:**
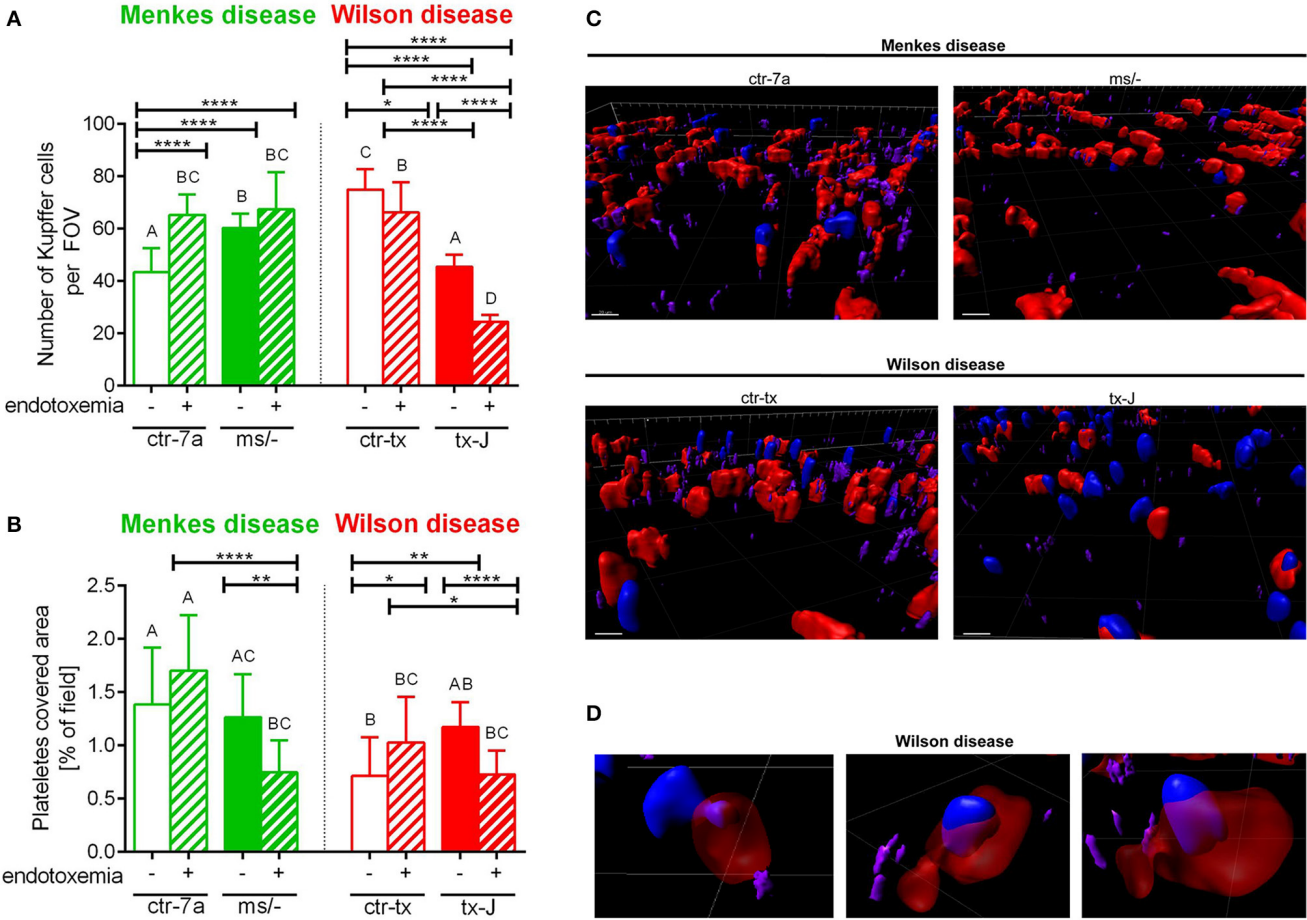
The presence of Kupffer cells **(A)** and platelets **(B)** in livers of Wilson (tx-J) and Menkes (^ms/−^) disease mice and their respective controls (ctr-tx and ctr-7a) with endotoxemia. Kupffer cells were counted per field of view (FOV), and area covered by platelets is expressed in percentage (as calculated by ImageJ) (mice *n* = 3/group). Asterisks indicate significant differences using unpaired two-tailed Student's *t* test (*P ≤ 0.05, **P ≤ 0.01, ****P ≤ 0.0001) and one-way ANOVA (*post hoc* Bonferroni) test (different letters indicate statistically significant differences between groups). A series of optical cross-sections (*z* stacks) were made through inflamed livers. Exemplary images of *z* stacks made through the liver of Menkes disease and Wilson disease mice and their respective controls are shown in **(C)**. Kupffer cells are marked in red, neutrophils in blue, and neutrophil elastase in violet. In endotoxemic mice with Wilson disease, neutrophils present (partially or fully) inside some Kupffer cells were observed **(D)**. The scale bar indicates 20 μm.

In the course of endotoxemia, accumulation of neutrophils in the liver is observed (7), and this phenomenon was also captured herein in livers of LPS-treated C57Bl/6J (not shown), and ctr-7a and ctr-tx mice (Figures 3A,B). However, in the case of Menkes disease mice, neutrophil numbers were as low as in ctr-7a untreated animals even after LPS administration. ^*ms*^/^−^ animals are in general characterized by low numbers of neutrophils as there are abnormalities in their maturation (25). On the contrary, neutrophil numbers in livers of tx-J mice with systemic inflammation were even higher than in endotoxemic ctr-tx animals (Figures 3A,B, 4C and Supplementary Movies 1, 2). On 3D *z* stacks performed through the liver, we also noticed that in Wilson disease mice, partially engulfed neutrophils were present in multiple KCs (Figure 4D). Although some neutrophils could also be spotted inside liver macrophages in Menkes disease mice, such events were rare.

### KC Counts in Menkes and Wilson Disease Mice During Endotoxemia

KCs constitute the largest population of resident macrophages in the body, and their primary function is to protect the liver from bacterial infections (41). As numbers of KCs might be strain-specific (42), we firstly verified their numbers in healthy mice and observed more KCs in C3HeB/FeJ (ctr-tx) than in outbred (ctr-7a) mice (Figure 4A and Supplementary Figure 2). However, Menkes mutants had more KCs than ctr-7a and Wilson disease animals. In the course of systemic inflammation, an increase in counts of KCs was observed, in both ctr-7a (statistically significant) and mutated ^*ms*^/^−^ animals (tendency) (Figure 4A). This was not a case either for ctr-tx or tx-J mice, and furthermore, these animals had significantly less KCs present in their liver sinusoids 24 h post endotoxemia induction (Figures 4A,C).

### Platelet Accumulation in Sinusoids of Mice With Wilson and Menkes Diseases

Platelets are pivotal during systemic inflammation as they are important for a cross-talk between KCs and pathogens (43), and they might be important for NET formation (44), and contribute to NET pathology (10). We detected less platelets in sinusoids of ctr-tx (C3HeB/FeJ) mice than the outbreds, which is in line with reported variations between some murine strains (45). However, there were no differences between the control animals and their respective mutants (Figure 4B). According to ANOVA, there were no significant differences in platelet accumulation in liver sinusoids of Wilson disease mice (tx-J) and their littermates, although *t* test showed weaker platelet accumulation upon endotoxemia. The same pattern was observed for Menkes disease mice (Figure 4B).

### Functional Characteristics of Neutrophils Isolated From Mutant Mice

**Figure 5:**
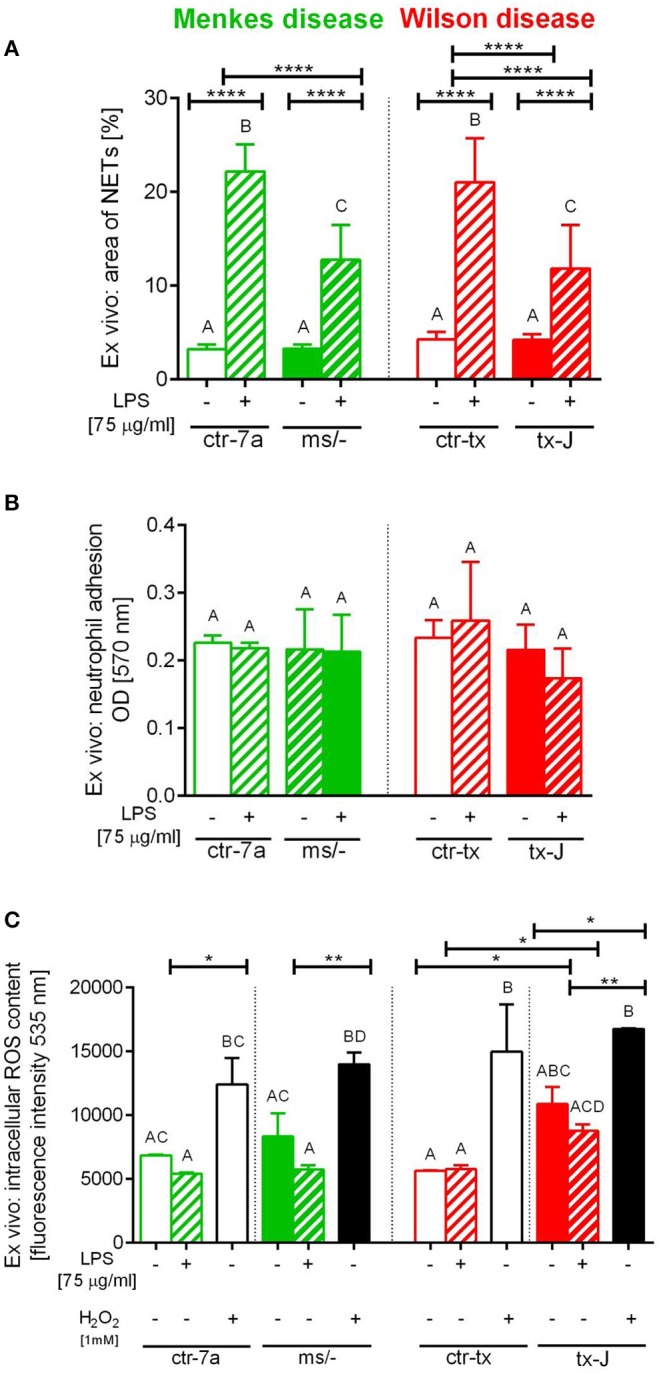
*Ex vivo* studies on isolated bone marrow neutrophils of Wilson (tx-J) and Menkes (^ms/−^) disease mice and their respective controls (ctr-tx and ctr-7a) evaluating their capacity to form NETs **(A)**, adhere **(B)**, and release reactive oxygen species (ROS; **C**). NET formation and capacity to adhere were evaluated 6 h after stimulation with LPS, and the content of ROS was measured after the first hour (DCF-DA test). In **(C)**, white/black bars represent responses of cells treated with H_2_O_2_ (positive control)—data for neutrophils from control mice (white bars) and mutants (black bars). Asterisks indicate significant differences using unpaired two-tailed Student's *t* test (*P ≤ 0.05, **P ≤ 0.01, ****P ≤ 0.0001) and one-way ANOVA (*post hoc* Bonferroni) test (different letters indicate statistically significant differences between groups).

To verify if reduced NET formation detected during systemic inflammation in either ^ms^/^−^ or tx-J mice resulted from intrinsic restrains of neutrophils or rather the microenvironment, basic parameters of neutrophils were evaluated (Figure 5). The viability of neutrophils isolated from all mutant and control strains was similar (data not shown) and so was their ability to adhere (Figure 5B). The only difference was stronger adhesion of neutrophils from mice on C3HeB/FeJ background (both ctr-tx and tx-J) detected 3 h after cell seeding, suggesting that this was due to genetic factors (data now shown). However, after 6 h, there were neither intergroup nor interstrain differences in neutrophil adhesion (Figure 5B). We also did not detect differences in the capacity to perform respiratory burst/release ROS between Menkes disease mice and their genetically unaltered littermates (Figure 5C and Supplementary Figure 3). However, mice on the outbred background (both ctr-7a and ^ms^/^−^) started producing more ROS after several hours (6 vs. 1–3 h; Supplementary Figure 3B vs. Supplementary Figure 3A, Figure 5C, respectively), whereas a reversed pattern was observed in mice on the C3HeB/FeJ background. Importantly, we detected that neutrophils isolated from both mutant strains could form NETs upon LPS stimulation *ex vivo* although they released less NETs than their unmutated controls (Figure 5A).

### Phenotype of Copper Chelator Treated Mice

**Figure 6:**
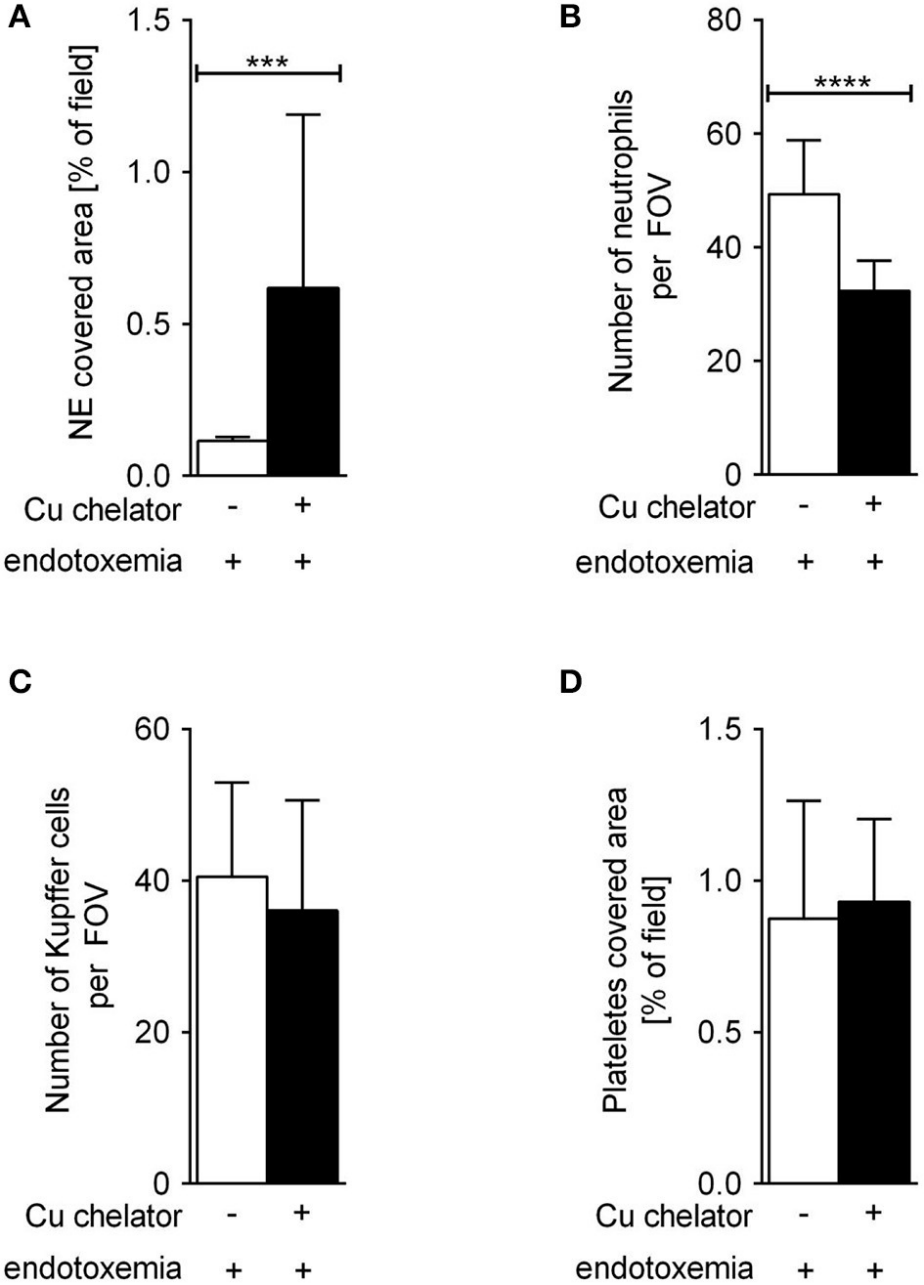
The impact of subchronic copper deficiency on NET formation **(A)**, neutrophil numbers **(B)**, Kupffer cell counts **(C)**, and platelet covered area **(D)** in C57Bl/6J mice. Copper chelator (Cu chelator) was administered for 8 days and then LPS-systemic inflammation was induced. NET covered area and cell counts were performed 24 h later. Asterisks indicate significant differences using unpaired two-tailed Student's *t* test (***P ≤ 0.001, ****P ≤ 0.0001).

Some C57Bl/6J mice were treated with a Cu chelator for eight constitutive days and then treated with LPS to induce 24-h systemic inflammation. In these animals, liver sinusoids were layered with significantly more NETs than in their saline-treated counterparts (Figure 6). Albeit the numbers of KCs (Figure 6C) and platelets (Figure 6D) were unchanged in these mice, significantly less neutrophils infiltrated liver sinusoids (Figure 6B).

### Copper Levels in Mice With Wilson and Menkes Diseases

**Figure 7:**
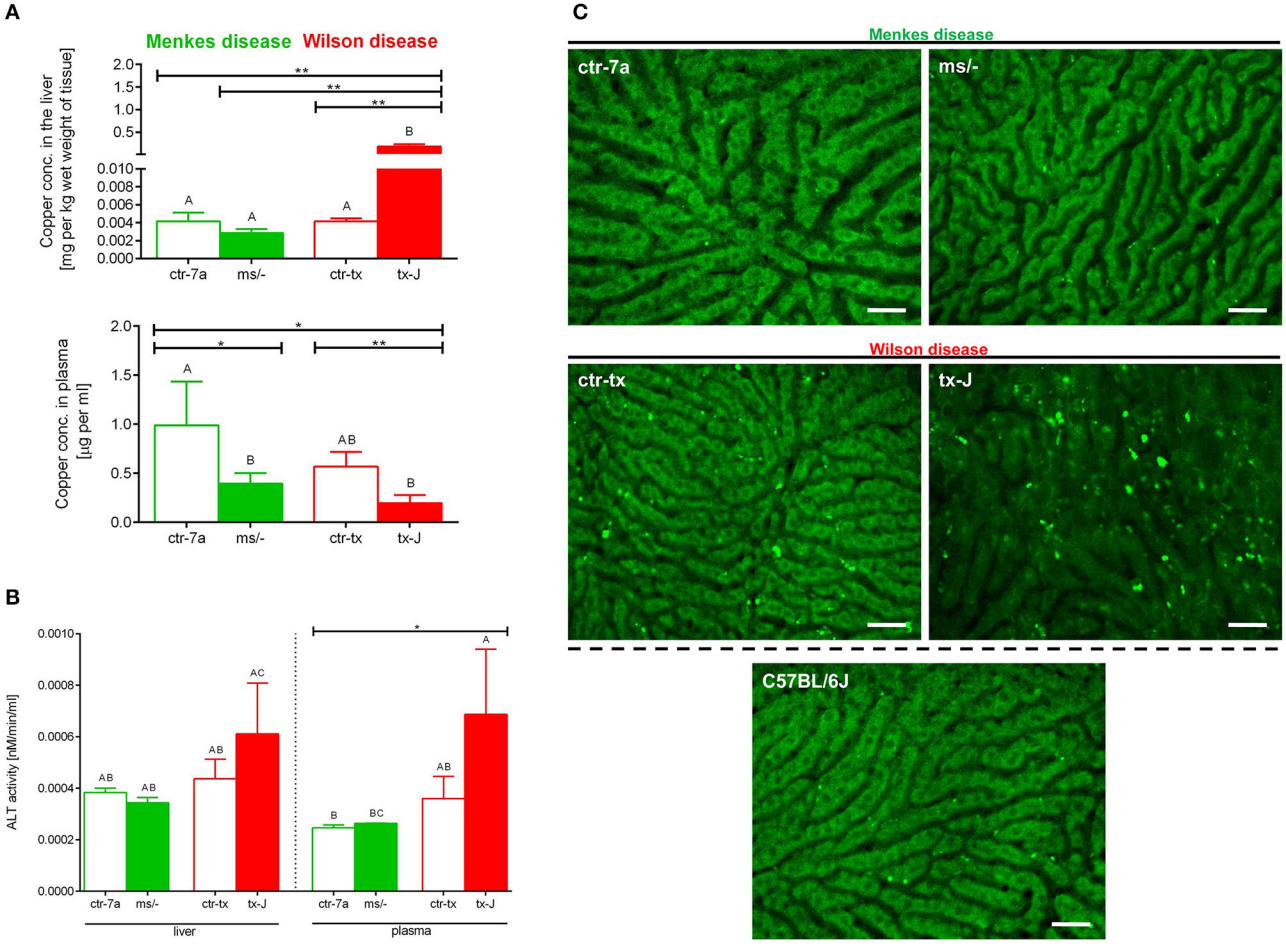
Liver and plasma copper accumulation **(A)** and alanine transaminase (ALT; **B**) levels estimated in mice with Wilson (tx-J) and Menkes (^ms/−^) diseases and their respective controls (ctr-tx and ctr-7a). Asterisks indicate significant differences using unpaired two-tailed Student's *t* test (*P ≤ 0.05, **P ≤ 0.01) and one-way ANOVA (*post hoc* Bonferroni) test (different letters indicate statistically significant differences between groups). Condition of hepatocytes, visibility of sinusoids, and deposition of autofluorescent aggregates is shown on representative images of livers in **(C)**; images of mice with Wilson and Menkes diseases are shown along with their controls and C57Bl/6J mice. No additional staining was performed, and only autofluorescent hepatocytes (green) were recorded in the GFP channel. Between the hepatocytes, sinusoids (black ducts) can be observed. The scale bar indicates 50 μm.

Copper levels were estimated by AAS. Despite Cu injections into mice with Menkes disease (^*ms*^/^−^), to keep them alive, their copper levels were still diminished in both the liver and (especially) the plasma (Figure 7A). As anticipated, we detected significant copper accumulation in livers of mice with Wilson disease (tx-J), higher by ~50-fold than in ctr-tx animals (Figure 7A). However, plasma copper levels were not increased in tx-J mice. In spite of being a disease of copper intracellular overload, the total serum copper is usually low/reduced in the circulation of individuals with the Wilson disease, mice or human, but it might increase in time (30, 46). This is in contrast to copper levels in the liver and the GI tract where its high accumulation impacts cells, including infiltrating leukocytes.

### Liver Damage in Mice With Wilson Disease vs. Menkes Disease

Alanine aminotransferase (ALT) can be found in the blood and some organs, although in greatest abundance in the liver, and its increased levels/activity are indicative of liver damage (7). Activity of ALT was unchanged in Menkes disease mice as measured in their liver homogenates and plasma (Figure 7B). Conversely, there was a tendency to increased ALT activity in both liver tissue and plasma of mice with Wilson disease, but the differences did not reach statistical significance (Figure 7B). The data corresponded with the architecture of liver sinusoids; i.e., while livers appeared to emit a homogeneous autofluorescent signal in Menkes disease mice, sinusoids were well-visible and hepatocyte nuclei were clearly observable; in Wilson disease mice, all these parameters were altered (Figure 7C, representative images; Supplementary Movies 1, 2). Hepatocyte morphology was disturbed, their nuclei were hardly evident, and sinusoids were less visible and less of them appeared on average in FOVs. Moreover, numerous green autofluorescent corpuscles were observed (Figure 7C and Supplementary Movies 1, 2). It should be stressed that some autofluorescent aggregates, although much less frequent, were also detected in controls of Wilson disease mice (ctr-tx).

### Expression of CTR1, ATP7A, and ATP7B in Neutrophils

One of the cellular copper transporters is high-affinity copper uptake protein 1 (CTR1), and its expression was detected in both resting neutrophils and those incubated with copper (0.5, 1, and 2 μg/ml) whereas the signal was stronger (more dispersed) in cells cultured without it (Supplementary Figure 4). This is in agreement with CTR1 expression in other organs of mice exposed to either copper-rich or -deficient diets (47). Nevertheless, the expression of CTR1 was detected only on up to 5% of cells (data not shown), which suggests that other copper transporter(s) must also be operating in neutrophils. In line with this, divalent metal transporter 1 (DMT1) was shown to be upregulated on inflammatory neutrophils (42). Also the presence of ATP7A and ATP7B proteins was detected in some neutrophils (~5%) either resting or incubated with copper (Supplementary Figure 4). ATP7A expression seemed dispersed between the trans-Golgi network and through the cytoplasm, independently of the presence of exogenous copper (Supplementary Figure 4). This is in line with ATP7A shuttling copper between the Golgi apparatus and the cell membrane to maintain its proper concentration (21). However, while ATP7B expression was also diffused throughout the cytosol in resting cells, it was more confined to Golgi apparatus in the presence of copper (Supplementary Figure 4).

### *Ex vivo* Studies: Viability of Neutrophils and Formation of NETs in the Presence of Exogenous Copper

**Figure 8:**
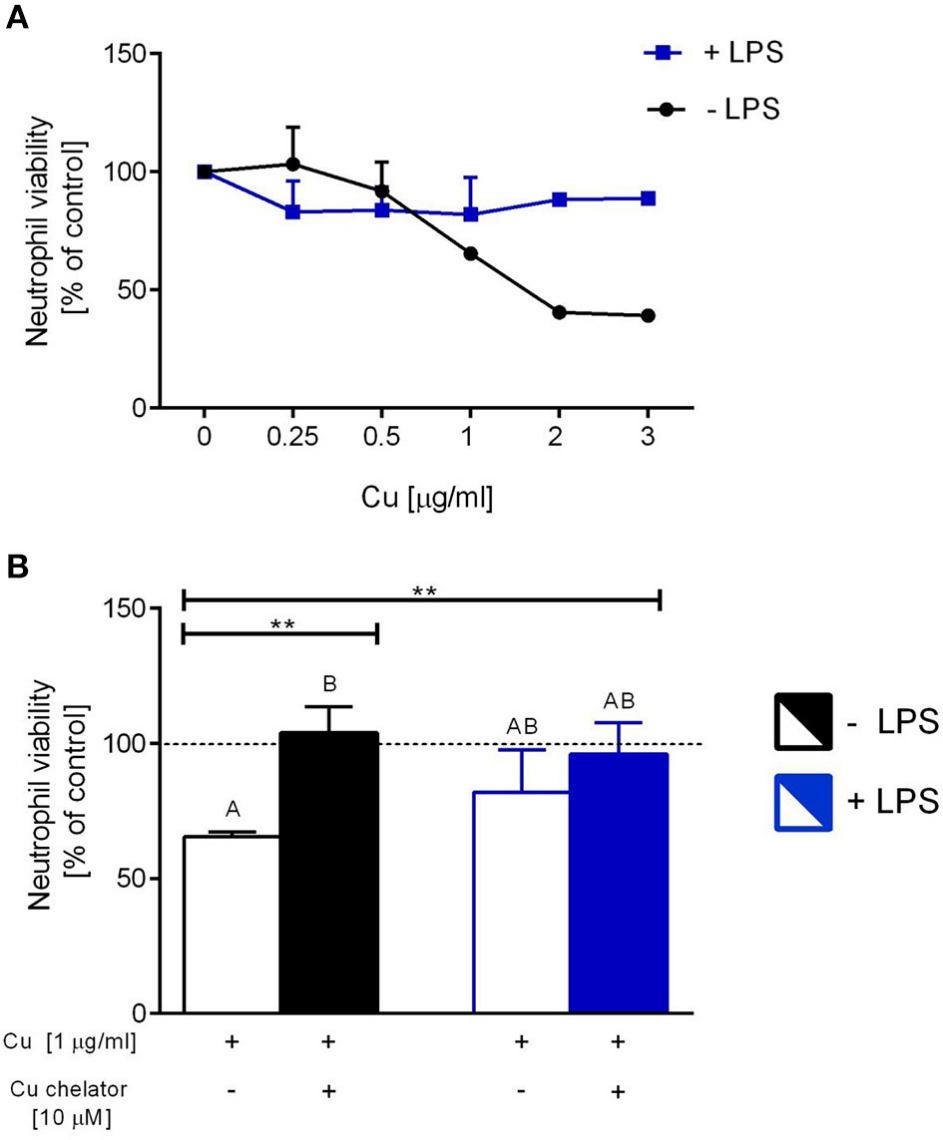
The impact of exogenous copper of neutrophil viability **(A)**, and counteraction of copper effects by its chelating agent **(B)** in *ex vivo* conditions. Neutrophils isolated from the bone marrow of C57Bl/6J mice were incubated with various copper concentrations in the presence (+LPS) or absence (–LPS) of lipopolysaccharide **(A)**. Cell viability reversed by the Cu chelator **(B)**. The dotted line **(B)** indicates values for untreated neutrophils (Cu concentration “0” in **A**). Asterisks indicate significant differences using unpaired two-tailed Student's *t* test (**P ≤ 0.01) and one-way ANOVA (*post hoc* Bonferroni) test (different letters indicate statistically significant differences between groups).

**Figure 9:**
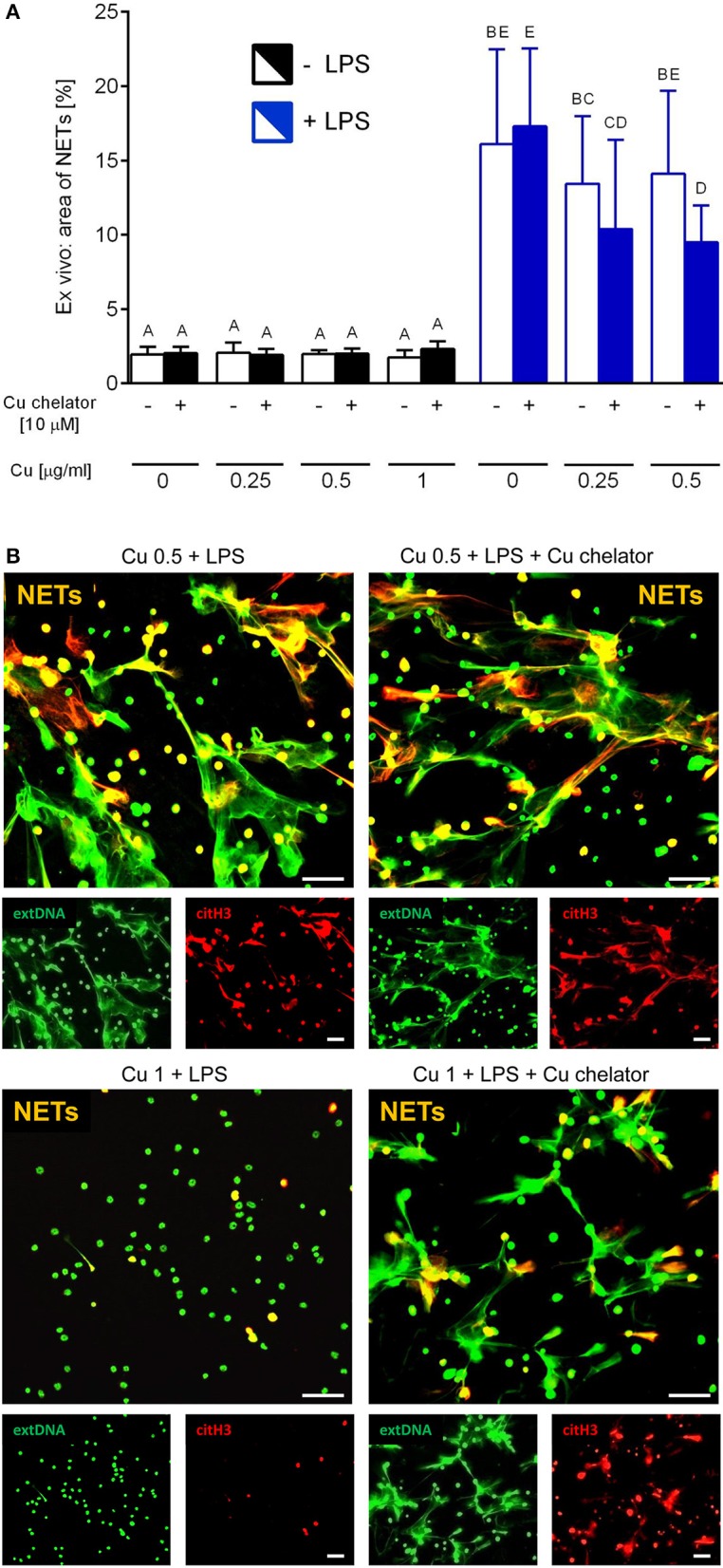
Neutrophil extracellular trap (NET) formation in the presence of exogenous copper. Neutrophils were either incubated with LPS (+LPS), various concentrations of copper (Cu), or left alone. Additionally, some cells were stimulated with LPS and/or copper chelator when co-cultured with the varying concentrations of copper. In all groups, neutrophil capacity to form NETs was quantified **(A)**. Different letters indicate statistically significant differences between groups using one-way ANOVA (*post hoc* Bonferroni). Representative images of NETs formed by LPS in the presence of copper with or w/o copper chelator are shown in **(B)**. Extracellular DNA (extDNA) is shown in green and citrullinated histone H3 (citH3) is shown in red. To visualize co-localization of NET components, the images from each channel were overlaid (NETs, overlay; orange). The scale bar indicates 50 μm.

Isolated neutrophils, when treated with various concentrations of copper, revealed high sensitivity to this element. Decreased viability of the cells was observed from concentration 1 μg/ml onwards when Cu was the only exogenous factor (Figure 8A; –LPS). When higher concentrations of copper were added to neutrophil cultures (up to 200 μg/ml), 100% mortality was observed (not shown). When neutrophils were additionally incubated with LPS, exogenous copper was less toxic (Figure 8A; +LPS). Copper chelator (TTM) rescued neutrophil viability in all concentrations of copper tested; here, shown for 1 μg/ml (Figure 8B; –LPS). Both LPS (Supplementary Figure 5) and PMA (not shown) alone induced NET formation while copper alone did not induce NET formation (Supplementary Figure 5). When NET release was induced by LPS in the presence of copper, it did not affect NET release in concentrations up to 1 μg/ml, and neither did copper chelator (Figure 9A). Furthermore, the chelator restored NET formation by neutrophils incubated with 1 μg/ml of copper (Figures 9A,B).

## Discussion

Sepsis is one of the leading causes of death (48) and also patients with endotoxemia are at risk of increased mortality (11). Moreover, endotoxemia occurs in many patients with sepsis, but also in many clinical settings that are non-infectious in nature. A severe form of systemic inflammation is characterized by (multi)organ failure and although liver is not the most commonly affected organ, when it is, this becomes a grave complication and leads to its acute failure (12). Moreover, LPS plays a prominent role in liver injury in rodents and human individuals (49). We furthermore confirmed these findings in mouse models of bacterial and LPS-induced systemic inflammation and we were able to correlate them with NET formation and their inadequate removal, proteolytic activity of neutrophil elastase, and microcoagulation connected with platelet deposition (7, 10). While knowledge on direct NET inducers is rather vast, we still do not know much about microenvironmental factors that are critical for casting the traps. Involvement of some trace elements in NET formation was studied previously, and it was shown that formation of PMA-induced NETs is zinc dependent (2), but impact of iron is less clear as various iron-chelating agents were shown to either stimulate or inhibit NET formation (3, 4). Moreover, influx of calcium ions was shown to proceed NET release in numerous models (50). All the above elements were shown to be released (themselves or as a part of protein complexes) during NET formation, and the traps were also decorated with them (20). Also copper is present in neutrophils and was localized both in their cytoplasm and nucleus, and it is released from them in response to at least some of the NET inducers (PMA) (20, 51).

Herein we report that in mice with either phenotype, of high (Wilson disease) and low (Menkes disease) copper levels in the liver, NET formation during endotoxemia was impaired whereas NET release during this condition is one of the hallmarks of the human reaction to LPS and has been proposed to serve as its promising blood biomarker (6, 52). The fact that less NETs were formed in inflamed livers of Menkes mice correlates with hardly occurring neutrophil infiltration and impaired neutropoiesis is most probably directly responsible for this phenomenon. Of note, we also show that respective controls of Wilson/Menkes disease mice produce significantly less NETs than C57Bl/6J mice, with C3HeB/FeJ mice displaying the weakest capacity to cast NETs. Thus, these data further extend previous findings on murine strain differences in respect to the efficiency of NET formation (53). In animals with Wilson disease (*Atp7b* mutants), indeed very high accumulation of copper was observed in the organ and accordingly liver was injured. In particular, the appearance of the organ revealed with intravital microscopy was striking as sinusoids were hardly visible, and hepatocytes were enlarged and their nuclei were barely evident, a phenotype coinciding with hematoxylin-stained liver sections (33). Moreover, numerous autofluorescent bodies could be detected. Autofluorescence and its distribution in the liver is an intrinsic parameter that can provide real-time information on the morphology and functional properties of this organ (54). When liver is malfunctioning, autofluorescence is more prominent and rather than by NAD(P)H, it is emitted by collagen and vitamin A (54).

In damaged (non-infectious) liver, NET formation is very weak as we showed previously for sterile thermal liver injury and further compared it to systemic bacterial infection (55). However, although very few NETs were observed during systemic inflammation in Wilson mice, at the same time, a larger number of neutrophils infiltrated the liver than in endotoxemic control (ctr-tx) animals. This indicates that the cells were not prone to cast NETs. To verify it further, we performed an *ex vivo* experiment in which we incubated neutrophils isolated from bone marrow with various copper concentrations. Our first observation was that neutrophils were very sensitive to copper and they remained fully viable only in copper (II) chloride concentrations of up to 0.5 μg/ml. This is in line with the fact that ~0.5 μg/ml is an average serum concentration of copper in healthy C57Bl/6J mice (56) and mice on the C3HeB/FeJ background (Figure 7A; ctr-tx). However, in the presence of LPS, neutrophils were less sensitive to copper. This might be connected to the fact that neutrophils activated by pro-inflammatory factors such as LPS have a prolonged life span and delayed apoptosis (57). Moreover, one of the effects of transition metals, including copper, on cells is induction of apoptosis. In fact, Cu-induced hepatotoxicity and neurotoxicity, occurring also during Wilson disease, is connected to its pro-apoptotic action (58).

Neutrophils primed *ex vivo* to cast NETs by LPS were releasing their similar quantities in the presence of exogenous copper within the physiological range (0.25–0.5 μg/ml) and copper-chelating agent only slightly (if at all) decreased the process. However, at the copper concentrations that were toxic to neutrophils (>1 μg/ml) significantly less NETs were released and the copper chelator restored this phenomenon. This indicates that indeed high copper concentrations prevent the release of NETs or it directly echoes low numbers of live neutrophils.

To verify if neutrophils themselves are equipped with the machinery to control intracellular copper levels, we studied cellular expression of some of its regulators. We revealed that at least some neutrophils express the copper transporter — CTR1, and moreover some of them express both ATP7A and ATP7B (up to 5% of neutrophils). While expression of ATP7A is anticipated in most cell types (21), ATP7B expression was so far only reported in hepatocytes and KCs (33). To the best of our knowledge, expression of ATP7 isoforms was not studied in neutrophils so far. Since neutrophils from Wilson disease mice lack functional ATP7B, they might still be able to efflux excessive copper via ATP7A. But our studies indicate that only to a certain point beyond which the cells first decrease their activity (weaker NET release at 1 μg/ml) and then die (>1 μg/ml). In fact, the latter concentration is the one observed in the serum of ATP7B-deficient mice at the age of 28 weeks and older (30). Interestingly, transplantation of only hepatocytes of ATP7B-deficient mice into spleens of their control littermates increases serum copper levels by 60% (59). Overall, the above studies indicate that the production of neutrophils is not diminished by high copper concentrations (serum and liver), and during systemic inflammation, these cells infiltrate liver even stronger than in genetically wild type mice. This might suggest that higher numbers of neutrophils are recruited to the liver to compensate for lower neutrophil activity.

In Wilson disease mice, also lower numbers of KCs and platelets were detected than in their control counterparts. KCs are macrophages of the liver that are central to both the hepatic and systemic response to infection (60). As they normally express ATP7B to regulate their copper levels, they are as overloaded with the element as hepatocytes (33), which most probably results in their death. In fact, KCs cooperate with platelets during the early stages of systemic inflammation as platelets facilitate a cross-talk between KCs and pathogens/their derivatives (43). Because numbers of KCs are lower in Wilson disease mice, platelet counts might be diminished as they adhere to KCs in the course of endotoxemia (43). Even more importantly, during ongoing sepsis, profound platelet aggregation occurs within and around NETs, and it is significantly diminished when they are removed or not formed (10). We propose the above factors to be responsible for weak platelet deposition in inflamed livers of Wilson disease mice. Interestingly, we also noticed neutrophils present inside numerous KCs of Wilson disease mice. This might suggest that more neutrophils die while present in the copper overloaded liver and they are engulfed by liver macrophages. Additionally or alternatively, due to low numbers of KCs, dying neutrophils are not properly removed, which results in their accumulation in the organ.

In mice with the Menkes disease (*Atp7a* mutants) phenotype, also significantly less NETs were detected in the course of endotoxemia. However, in contrast to tx-J animals, in these mice, significantly less neutrophils were infiltrating the liver and it is known that copper deficiency impairs their maturation in the bone marrow (25). As mentioned above, KCs are important for the initiation of systemic inflammation as together with platelets they activate a proper inflammatory response that leads to neutrophil infiltration (43). During systemic reaction, their numbers might slightly increase (60–62), and this was also observed in mice with Menkes disease. However, although copper deficiency does not seem to impact numbers of KCs, it is known to affect some macrophage activities (e.g., their capacity to perform respiratory burst) (63). Moreover, reduced superoxide anion production was also observed in neutrophils of copper deficient individuals (64). Therefore, lower levels of NETs released during endotoxemia in *Atp7a* mutant mice might have also resulted from weaker production of ROS. However, NET formation was shown to be ROS(NOX)-independent in multiple settings including sepsis, and so was NET release induced by complement receptors, TLR2/TLR4 ligands or TLR4-activated platelets (7, 40, 65). In fact, physiological NET triggers (pathogens and their derivatives, and cytokines) seem to act in a ROS-independent manner (66). Therefore, weaker NET release probably rather results from low neutrophil numbers. The Menkes disease mice also had lower platelet counts present in inflamed livers. Alteration in platelet capacity to adhere and form plug/thrombus was shown previously in copper-deficient animals (67, 68) but what we observed in the current study might simply result from less NETs being cast as they form a platform for platelet adhesion in LPS-induced systemic inflammation (10).

To verify if copper deficiency results in a weaker NET formation, we kept C57Bl/6J mice, with unaltered ATPase expression, on daily copper chelator injections for 8 days to mimic a semi-chronic copper deprivation. To our surprise, during endotoxemia, much stronger NET release was detected in the livers of these animals than in their saline-treated controls. Similarly as in *Atp7a* mutants, less neutrophils were present in liver sinusoids but the numbers of KCs and platelets were unchanged. These results imply that indeed copper deficiency directly affects neutrophil numbers but the impact on NET formation is more complex. In our *ex vivo* experiments, copper chelator alone (when no exogenous copper was added) was not impacting NET formation (even in higher concentrations than show here), suggesting that copper is not essential for the release of NETs. The fundamental difference between mice with subchronic copper deficiency and *Atp7a* mutated animals is high accumulation of copper in the intestines of the latter animals (35). It is because copper has been shown to affect microbiota diversity in the gut of mice (69) and microbiota content (both qualitative and quantitative) is critical for proper aging of neutrophils and their removal as well as for casting NETs (70). Furthermore, antibiotic therapy was shown to affect ATP7A expression in the colon of mice (71). Therefore, depending on the status of microbiota, diverse numbers of aged neutrophils might be available in mice with subchronic copper deficiency and *Atp7a* mutants, while it is the aged neutrophil phenotype that exhibits enhanced NET formation under inflammatory conditions (70). Although we are unaware of studies on microbiota composition in Menkes disease-affected humans or mice, it is known to be altered in Wilson disease patients (72). Therefore, verification of microbiota changes in Menkes patients and in Wilson and Menkes mice could shed light on this issue.

In conclusion, with the application of intravital microscopy, we revealed that in both *in vivo* phenotypes of inappropriate copper distribution/levels and efflux in the body, resulting from altered genotypes, NET formation is decreased during systemic inflammation. This implies that at early stages of the reaction, affected individuals are less armed to capture pathogens and indeed some Wilson disease patients are more prone to sepsis (73). Overall, the study reveals that the requirement for different microelements for NET formation considerably varies as unlike zinc, low or negligible levels of copper do not interfere with NET formation or might even enhance it. In contrast, high copper concentrations (beyond 1 μg/ml) inhibit NET release, but this is mostly due to the cytotoxicity toward neutrophils.

## Data Availability Statement

The datasets generated for this study are available on request to the corresponding author.

## Ethics Statement

All protocols were approved by the Local Ethical Committee No. II in Krakow (Permission Nos. 293/2017 and 293A/2018).

## Author Contributions

IC acquired data (performed all intravital microscopy, *ex vivo* studies, immunocytochemistry, biochemical tests), performed all analyses, and participated in interpretation of data. WO acquired data (some *ex vivo* studies, immunocytochemistry). ML and AB provided mice, ATP7Bα, and CTR1α, and secured ASS analyses. EK provided study conception and design, participated in analyses and interpretation of data, and wrote the manuscript.

### Conflict of Interest

The authors declare that the research was conducted in the absence of any commercial or financial relationships that could be construed as a potential conflict of interest.

## Supplementary Material

Supplementary Movie 1 Menkes disease mice (^ms/−^) and their controls (ctr-7a) with 24-h endotoxemia. Neutrophils were labeled with Brilliant Violet 421 anti-Ly6G antibody (blue), neutrophil elastase with Alexa Fluor 647 anti-NE (violet) and platelets with PE anti-CD49b (red). Autofluorescent hepatocytes are green. The scale bar indicates 50 μm.

Supplementary Movie 2 Wilson disease mice (tx-J) and their controls (ctr-tx) with 24-h endotoxemia. Neutrophils were labeled with Brilliant Violet 421 anti-Ly6G antibody (blue), neutrophil elastase with Alexa Fluor 647 anti-NE (violet), Kupffer cells with Alexa Fluor 488 anti-F4/80 (bright green) and platelets with PE anti-CD49b (red). Autofluorescent hepatocytes are green (dim green). The scale bar indicates 50 μm.
