# Supplementary material for: Reduced Neutrophil Extracellular Trap (NET) Formation During Systemic Inflammation in Mice With Menkes Disease and Wilson Disease: Copper Requirement for NET Release
Source: Front Immunol. 2020 Jan 15;10:3021. doi: 10.3389/fimmu.2019.03021 (PMC6974625; doi:10.3389/fimmu.2019.03021)

Supplementary Figure 1

Representative images of neutrophil extracellular trap (NET) formation in liver sinusoids of Menkes disease mice and their control littermates during endotoxemia (24 h post LPS). On images autofluorescent hepatocytes (green) can be observed; sinusoids are localized between them (black ducts). In the latter structures NE signal is deposited along endothelium (violet). UT – untreated. The scale bar indicates 50 μm

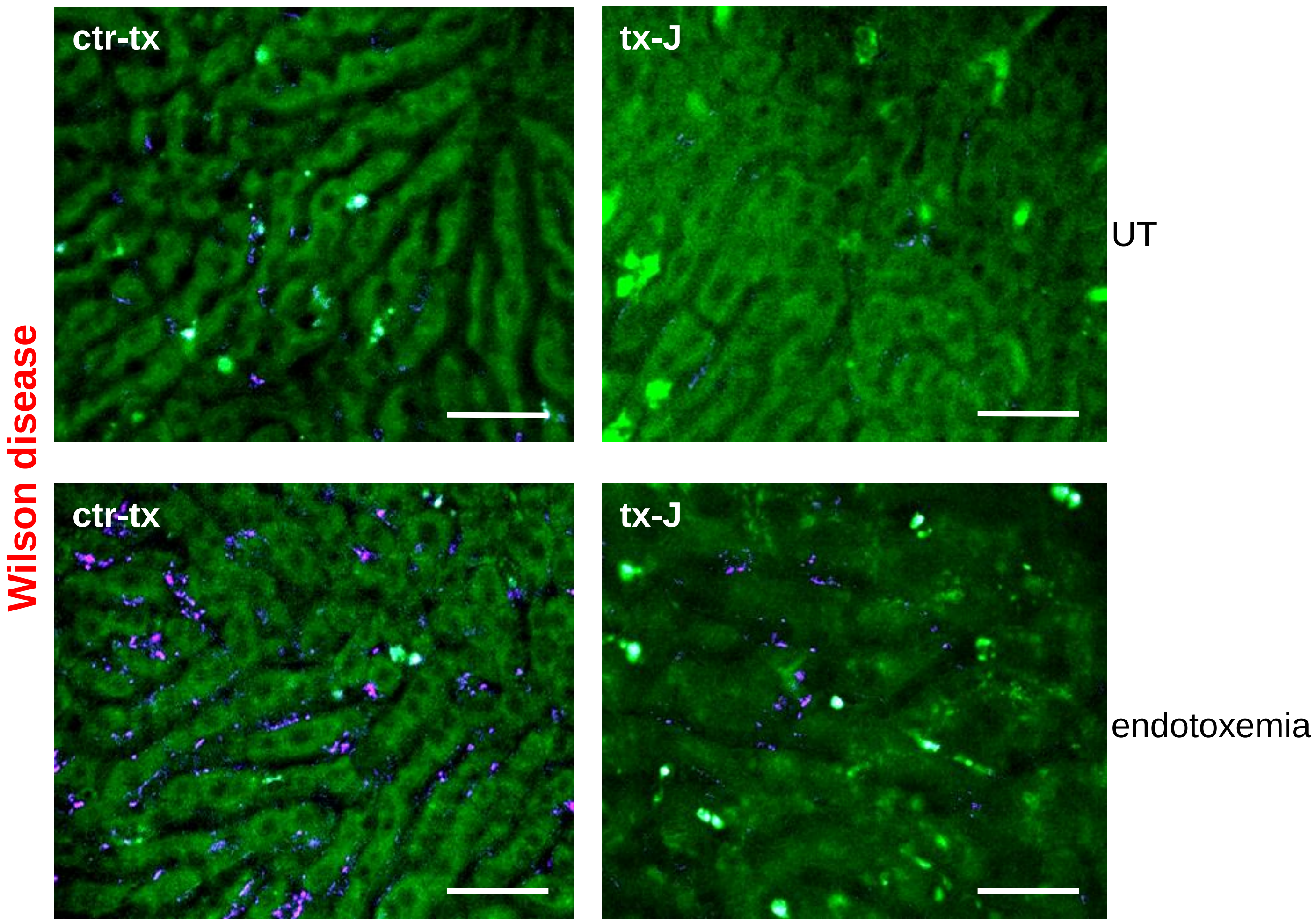

Supplementary Figure 2

Number of Kupffer cells within the liver sinusoids of Menkes and Wilson disease mice and their control littermates. The scale bar indicates 50 μm.

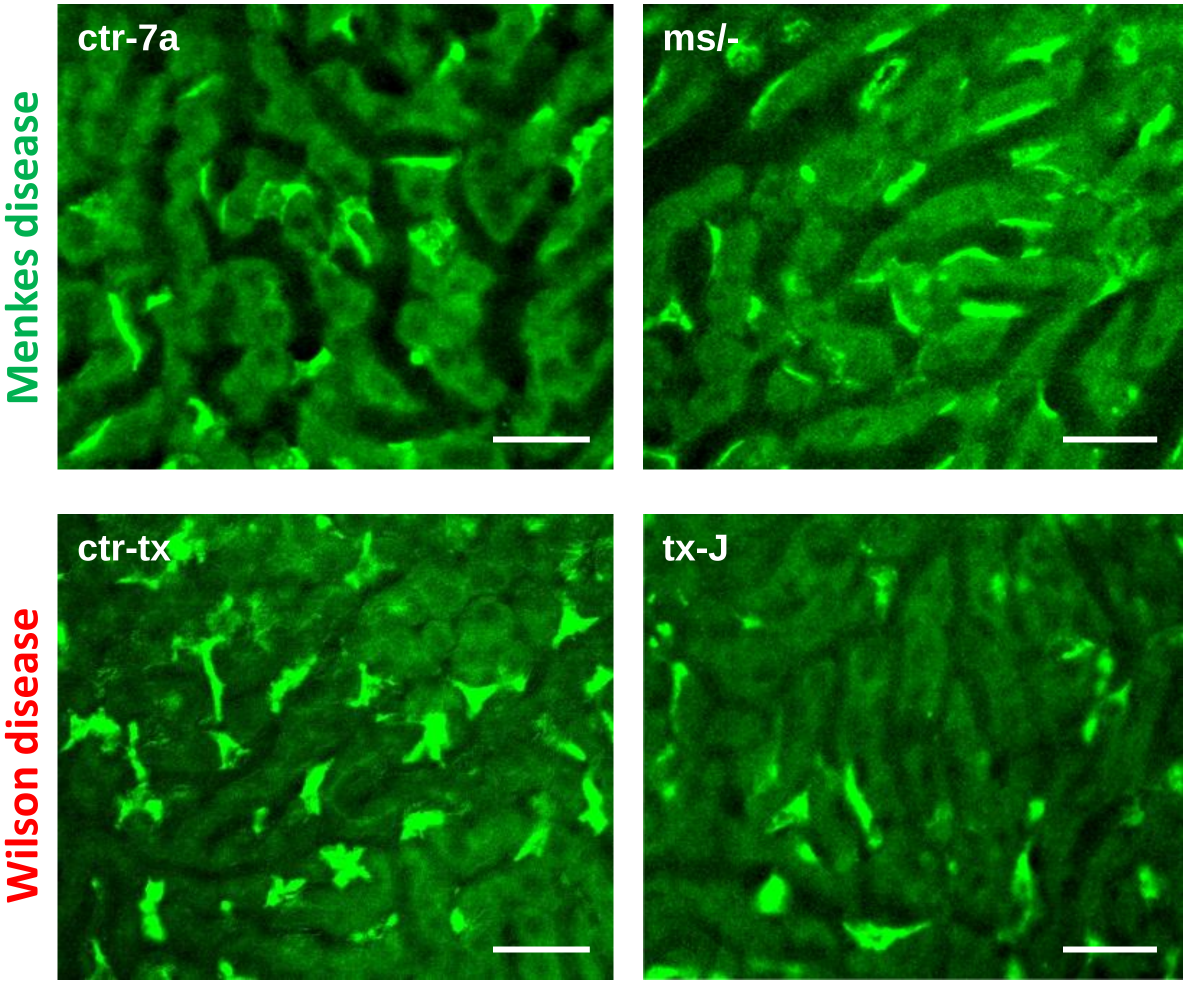

Supplementary Figure 3

Capacity of isolated neutrophils to perform respiratory burst as estimated by NBT test. Evaluation of ROS deposition in neutrophils was estimated 3 (A) and 6 (B) hours after stimulation with LPS. Different letters indicate statistically significant differences between groups (one-way ANOVA, post hoc Bonferroni test).

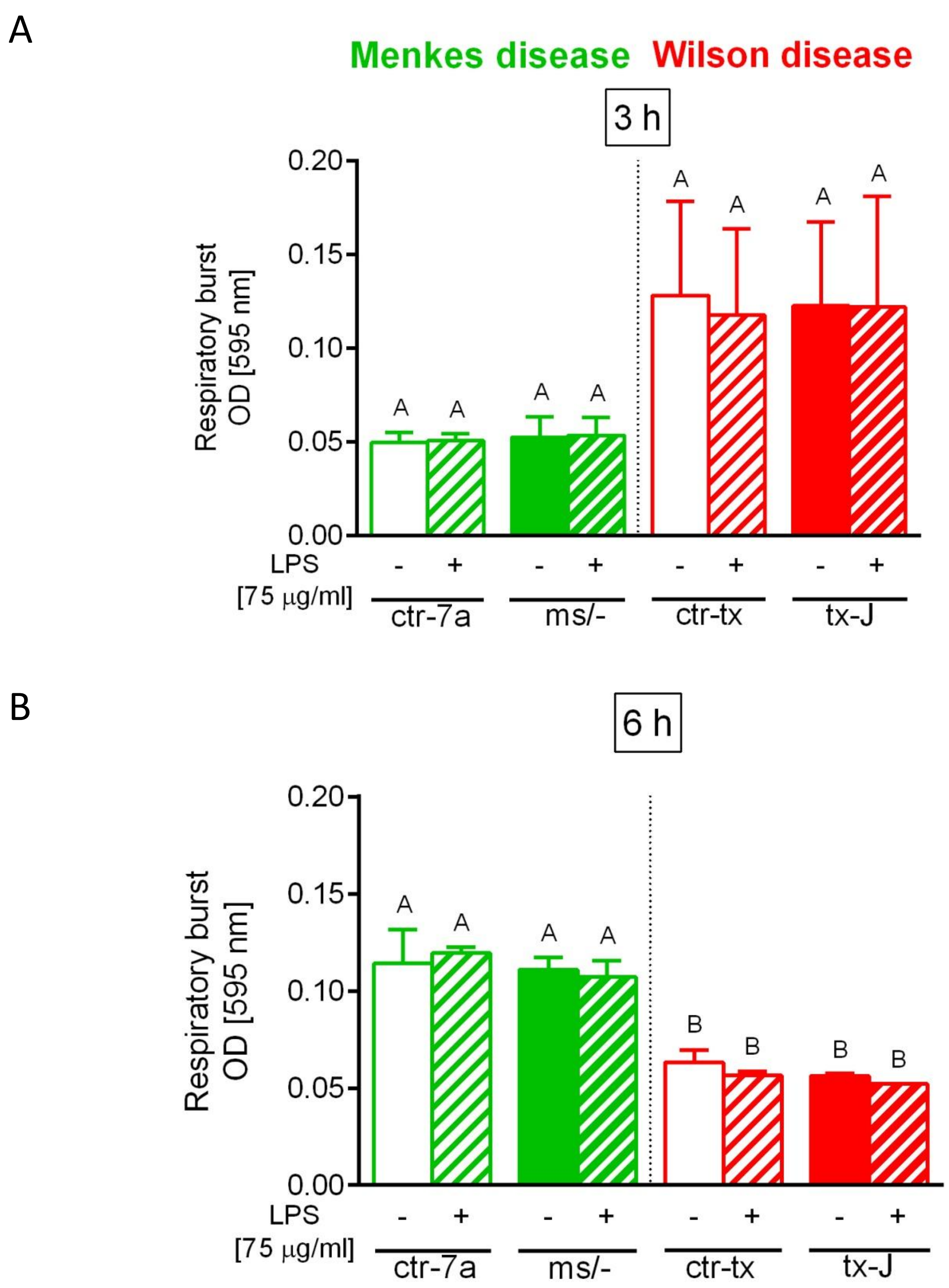

Supplementary Figure 4

Immunocytochemical cellular localization of CTR1, ATP7A and ATP7B (red) proteins in neutrophils of outbred mice. Note vesicular, cytoplasmic dispersed expression pattern of CTR1 and ATP7A in neutrophils incubated with copper (+Cu) versus in untreated cells (-Cu). Nuclei were counterstained with Sytox green. White dashed line denotes cells enlarged by 130% and marked with a yellow dashed line. The scale bar indicates 20 μm.

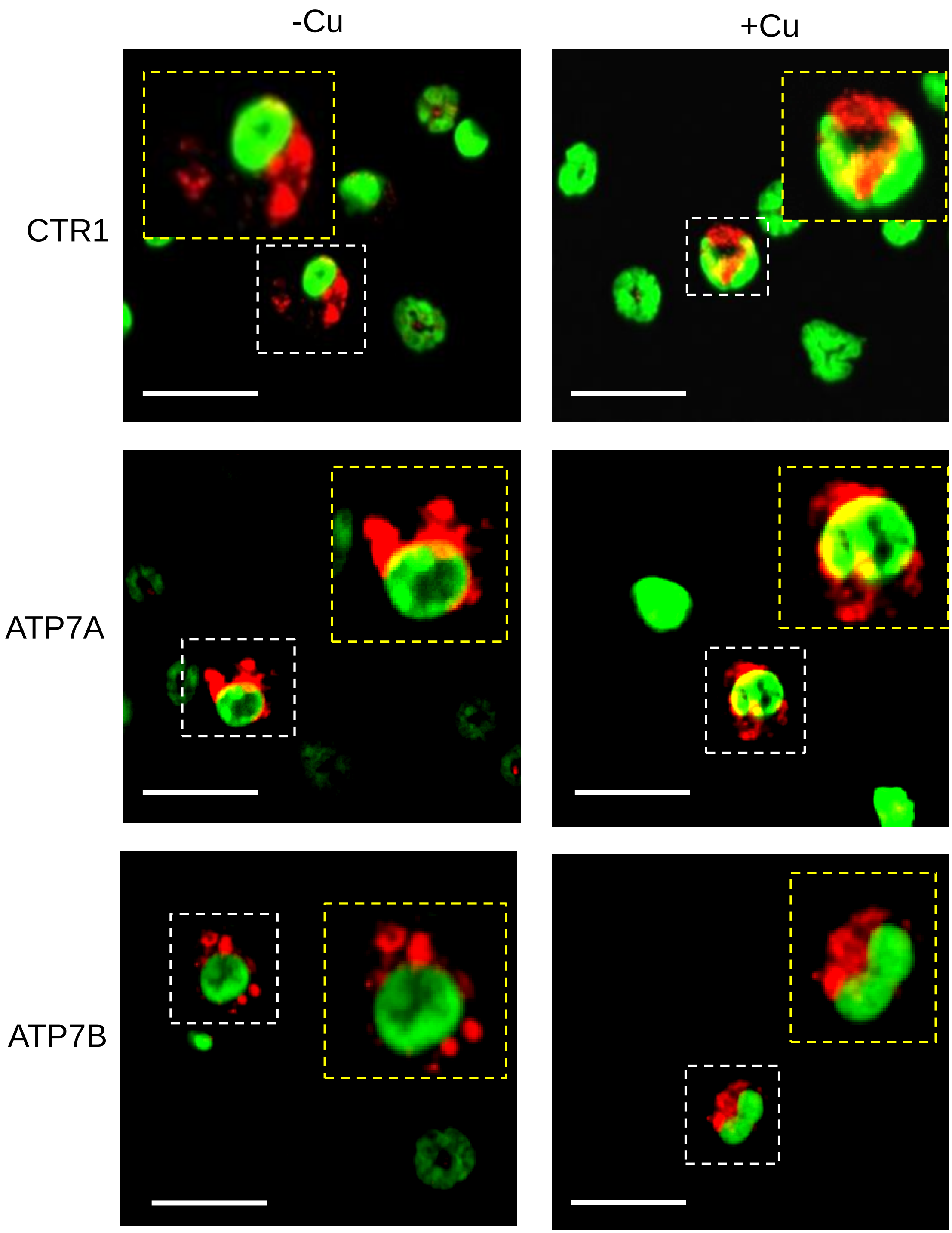

Supplementary Figure 5

Neutrophil extracellular traps (NETs) formation by isolated neutrophils in presence of either LPS (+LPS) or copper (+Cu; 0.5 µg/ml) alone. NETs were detected by immunocytochemistry and citrullinated histones (citH3) were stained in red while extracellular DNA (extDNA) in green. The scale bar indicates 50 µm.

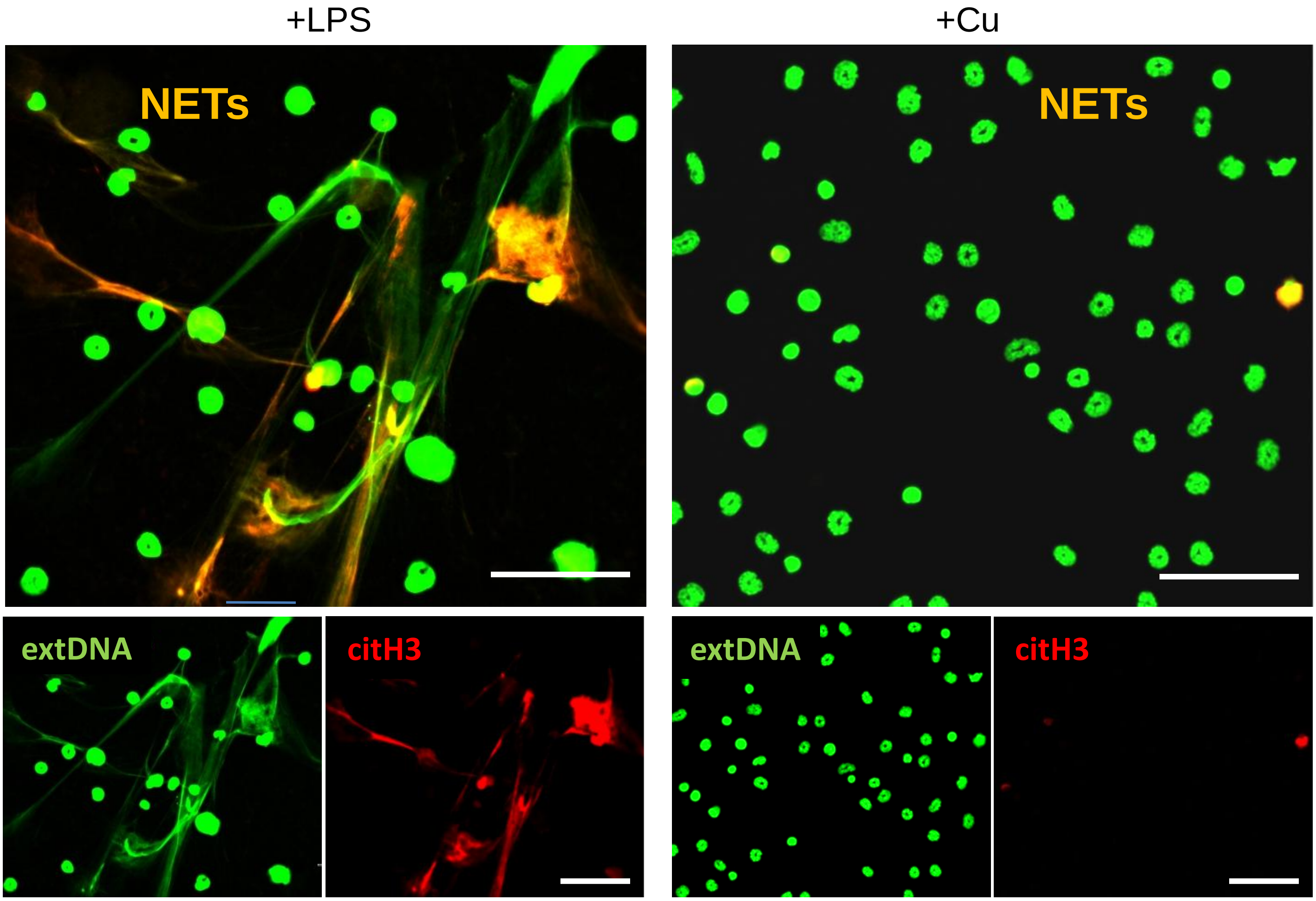

Supplement: Supplementary file 1 [file Presentation_1.pdf]
